# Computational Identification of Mechanistic Factors That Determine the Timing and Intensity of the Inflammatory Response

**DOI:** 10.1371/journal.pcbi.1004460

**Published:** 2015-12-03

**Authors:** Sridevi Nagaraja, Jaques Reifman, Alexander Y. Mitrophanov

**Affiliations:** Department of Defense Biotechnology High Performance Computing Software Applications Institute, Telemedicine and Advanced Technology Research Center, U.S. Army Medical Research and Materiel Command, Fort Detrick, Maryland, United States of America; University of Pittsburgh, UNITED STATES

## Abstract

Timely resolution of inflammation is critical for the restoration of homeostasis in injured or infected tissue. Chronic inflammation is often characterized by a persistent increase in the concentrations of inflammatory cells and molecular mediators, whose distinct amount and timing characteristics offer an opportunity to identify effective therapeutic regulatory targets. Here, we used our recently developed computational model of local inflammation to identify potential targets for molecular interventions and to investigate the effects of individual and combined inhibition of such targets. This was accomplished via the development and application of computational strategies involving the simulation and analysis of thousands of inflammatory scenarios. We found that modulation of macrophage influx and efflux is an effective potential strategy to regulate the amount of inflammatory cells and molecular mediators in both normal and chronic inflammatory scenarios. We identified three molecular mediators − tumor necrosis factor-α (TNF-α), transforming growth factor-β (TGF-β), and the chemokine CXCL8 − as potential molecular targets whose individual or combined inhibition may robustly regulate both the amount and timing properties of the kinetic trajectories for neutrophils and macrophages in chronic inflammation. Modulation of macrophage flux, as well as of the abundance of TNF-α, TGF-β, and CXCL8, may improve the resolution of chronic inflammation.

## Author Summary

A recent approach to quantitatively characterize the timing and intensity of the inflammatory response relies on the use of four quantities termed inflammation indices. The values of the inflammation indices may reflect the differences between normal and pathological inflammation, and may be used to gauge the effects of therapeutic interventions aimed to control inflammation. Yet, the specific inflammatory mechanisms that can be targeted to selectively control these indices remain unknown. Here, we developed and applied a computational strategy to identify potential target mechanisms to regulate such indices. We used our recently developed model of local inflammation to simulate thousands of inflammatory scenarios. We then subjected the corresponding inflammation index values to sensitivity and correlation analysis. We found that the inflammation indices may be significantly influenced by the macrophage influx and efflux rates, as well as by the degradation rates of three specific molecular mediators. These results suggested that the indices can be effectively regulated by individual or combined inhibition of those molecular mediators, which we confirmed by computational experiments. Taken together, our results highlight possible targets of therapeutic intervention that can be used to control both the timing and the intensity of the inflammatory response.

## Introduction

Prolonged inflammation is a recognized contributor to a multitude of pathological conditions, including cardiovascular, metabolic, and neurodegenerative diseases, as well as chronic injuries [1, 2]. Timely resolution of inflammation is essential for tissue homeostasis. Inflammation resolution, previously believed to be a passive, self-regulatory process, is now known to be actively modulated by several different classes of endogenous molecular mediators, such as anti-inflammatory cytokines and growth factors [interleukin-10 (IL-10) and transforming growth factor-β (TGF-β)], oxygenated lipid mediators (lipoxins, resolvins, protectins, and maresins), and protease inhibitors [2–5]. Ongoing research efforts, including pharmacological animal model research and clinical trials, are focused on novel pro-resolution therapies for a variety of inflammatory conditions [6, 7]. There is a clear, documented need for new approaches to develop resolution-centric therapeutic interventions [2] to supplement or replace the currently used anti-inflammatory therapies, which are only modestly effective [8–10].

**Fig 1:**
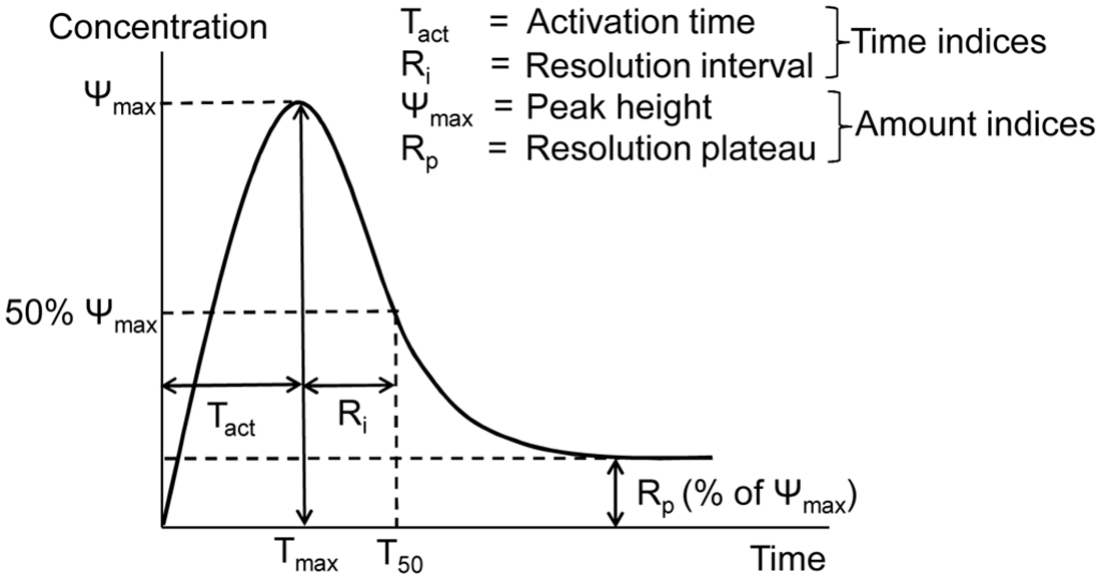
Quantitative indices of a typical inflammatory response curve. The timing indices (T_act_ and R_i_) and the amount indices (Ψ_max_ and R_p_) are defined as follows. Ψ_max_: peak height for a model output variable (molecular species or cell type); T_max_: time at which a model output variable reaches the peak height value; T_act_: time to reach peak height after inflammation initiation; T_50_: time for a model output variable to decay from its peak height value to 50% of the peak height; R_i_: difference between T_50_ and T_act_; R_p_: the value of a model output variable at the right-most time point of the simulation, as a percentage of the peak height value.

The concentrations of molecular and cellular components of the inflammatory process are typically characterized by single-peak temporal trajectories reflecting a distinct period of activation followed by resolution [2, 5, 11, 12] (Fig 1). The quantitative properties of these trajectories vary as a result of differences in inflammatory conditions and scenarios. Recently, four quantitative indices [namely, peak height (Ψ_max_), activation time (T_act_), resolution interval (R_i_), and resolution plateau (R_p_)] (Fig 1) were introduced as informative measures to analyze the quantitative patterns characterizing temporal inflammatory trajectories [1, 13]. For a given molecular species or cell type, the Ψ_max_ is defined as the maximum value of the corresponding temporal trajectory. T_act_ is the time after inflammation initiation required for the temporal trajectory to reach the Ψ_max_ level. R_i_ is the time difference between T_act_ and the time it takes to reach 50% of Ψ_max_. R_p_ is the trajectory level at the end of the considered time period, expressed as a percentage of the Ψ_max_ value; it therefore reflects residual inflammation. Based on the aspects of the temporal trajectories that they represent, the indices can be divided into amount indices (Ψ_max_ and R_p_) and timing indices (T_act_ and R_i_) [14, 15]. Additionally, the area under the curve (AUC) of a kinetic trajectory has been recently introduced as a metric to quantitatively assess cumulative tissue damage under various inflammatory scenarios [16].

Experimental studies have demonstrated the utility of the inflammation indices in distinguishing between the time courses for normal and pathological inflammation, as well as in establishing the quantitative efficacy of external interventions [1, 3, 17]. For example, in a mouse peritonitis model, it was shown that the macrophage Ψ_max_, T_act_, R_i_, and the neutrophil T_act_ were noticeably higher (specifically, higher by 2-fold, 3-fold, 2-fold, and 12-fold, respectively) in the chronic inflammatory scenario compared to the acute inflammatory scenario [17]. In the same study, several drugs (specifically, ibuprofen, resolvin E1, a prostaglandin D2 receptor 1 agonist, dexamethasone, rolipram, and azithromycin) were evaluated for their inflammation resolution efficacy by quantifying their regulation of these inflammation indices in the chronic inflammation scenario. Another peritonitis mouse model study investigated the efficacy of drug-filled nanoparticles in inflammation resolution. The drug efficacy was established based on its ability to reduce the neutrophil Ψ_max_ and T_act_ by ~1.5-fold [3]. Yet, systematic experimental characterization of the patterns and mutual relationships for the inflammation indices is challenging due to the immense diversity of possible inflammatory scenarios. This diversity hampers our understanding of the global control of the indices by specific molecular mechanisms, and our understanding of the modulation of the indices by therapeutic interventions. Computational modeling offers a possibility to complement experimental investigations and address this complex problem using an integrated approach.

Computational modeling can be used to “screen” thousands of inflammatory scenarios in a systematic way and thereby guide the generation of focused, mechanistic, experimentally testable hypotheses [11, 16, 18–21]. Previous works have demonstrated the utility of mathematical models in the study of inflammation in specific disease scenarios and in the identification of crucial inflammatory mechanisms [11, 12, 16, 20–32]. For example, computational models have been used to predict the efficacy of cytokine regulation therapies in chronic inflammatory diseases [24, 27, 33]. However, quantitative characterization of the inflammatory response of different cell types and molecular mediators in terms of indices, as well as a rigorous analysis of their mechanistic determinants, have not been carried out in previous modeling efforts. In the present study, we used our recently developed quantitative kinetic model of acute and chronic inflammation in wounds to generate hypotheses regarding mechanistic control of the inflammation indices [11]. Our model could capture the behavior of inflammatory cell type and molecular mediator kinetics in acute and chronic inflammation initiated by both infection and injury [17, 34, 35]. In our simulations, the chronic inflammatory scenarios were characterized by higher concentrations (Ψ_max_) and delayed resolution timing (R_i_) for key inflammatory components in comparison with acute inflammatory scenarios. Using this model, we identified essential inflammation-driving mechanisms (specifically, macrophage influx and efflux rates) and informative indicators (i.e., IL-6, TGF-β, and PDGF) of chronic inflammation. Our findings regarding the mechanistic regulation of inflammation by macrophage fluxes are supported by experimental studies where wound macrophage levels (regulated by macrophage fluxes) controlled the timing of wound healing [36–39] and the quality of wound scarring [40]. Furthermore, our modeling predictions characterizing IL-6 as an informative indicator of pathological inflammation are consistent with a recent clinical wound study [41]. Here, we use this model to elucidate the functional relationships between specific molecular/cellular processes and inflammation indices during normal and pathological inflammation.

The goal of our study was to identify specific mechanistic determinants that can be targeted to modulate the index values during abnormal (delayed) inflammation and drive them toward a desired outcome. We used two complementary analyses (namely, sensitivity and correlation analysis) and identified such targetable mechanisms for regulating the inflammatory indices in the model. Furthermore, we wanted to test the effectiveness of cytokine inhibition as an intervention strategy to regulate the inflammation indices. For this purpose, we extended the model to represent cytokine inhibition kinetics for three cytokines (chosen based on our predictions regarding targetable mechanisms). We used this extended model to study the efficacy of individual/combined cytokine inhibition in the regulation of the inflammation indices for neutrophils and macrophages.

Our modeling results indicate that, for the majority of the model output variables representing inflammatory cell types and molecular mediators, the amount indices Ψ_max_ and R_p_ were robustly regulated by the macrophage influx and efflux rate, respectively. In contrast, for the timing indices (i.e., T_act_ and R_i_), such a robust functional dependence on single model parameters was not detected in the sensitivity analysis. Yet, the timing indices for the inflammatory components were strongly correlated with the platelet degradation rate. Moreover, strong correlations between the timing indices and certain mechanistic processes existed, but only under specific inflammatory situations representing chronic inflammation. Our inflammatory mediator inhibition modeling suggested that during an abnormal (delayed) inflammatory response, TNF-α and TGF-β inhibition strongly shifted the macrophage Ψ_max_ and R_p_ indices toward inflammation resolution, whereas CXCL8 inhibition regulated the neutrophil R_i_ and could nearly restore this index to its normal (i.e., acute-inflammation) value. Notably, combined inhibition of TNF-α and CXCL8 resulted in improved restoration of normal neutrophil dynamics compared with the inhibition of these two targets acting independently. Comparisons with available experimental data provided validation for our TNF-α inhibitor modeling results.

## Results

### Model Parameters Effecting Robust Regulation of Inflammation Indices Identified via Sensitivity Analysis

To investigate the regulation of the inflammation indices via changes in the model parameters, we performed a local sensitivity analysis for the model in 10,000 inflammatory scenarios (simulations), as described in the Materials and Methods Section. We regarded the regulation of an inflammation index (computed for a given model output variable) by a specific parameter as *robust*, if the sensitivity *s*_*ij*_ (Eq 1, defined in the Materials and Methods Section) of the index with respect to this parameter was the highest (or second highest, third highest, etc.) across all parameters in the *majority* of the 10,000 simulations. In the figures and tables, for the sake of brevity, we identify each of the model’s 69 parameters using its assigned number in the model preceded by the prefix “P#.” We use these designations when referring to the figures and tables in the text and, in addition, provide descriptive names for the parameters (see Table 1 for a full list of the model parameters).

**Table 1:** List of model output variables (abbreviations) and model parameters with their assigned numbers (P#) and descriptions of their function [11].

Model output variables: Different cell types and molecular species			
*N*_*act*_	Active neutrophils	*TGFβ*	Transforming growth factor-β
*N*_*apop*_	Apoptotic neutrophils	*PDGF*	Platelet-derived growth factor
*M*_*pro*_	Pro-inflammatory macrophages	*IL*1*β*	Interleukin-1β
*M*_*anti*_	Anti-inflammatory macrophages	*IL*6	Interleukin-6
*P*	Platelets	*MIP*1*α*	Macrophage inflammatory protein-1α
*CXCL*8	Chemokine *CXCL*8	*MIP*2	Macrophage inflammatory protein-2
*IL*12	Interleukin-12	*IP*10	Interferon-γ-induced protein 10
*IL*10	Interleukin-10	*TNFα*	Tumor necrosis factor-α
*N*_*tot*_	Total neutrophils	*M*_*tot*_	Total macrophages
**P#**	**Model parameter description**		
1	Platelet degradation rate		
2	Rate of TGF-β release by platelets		
3	Chemotactic migration of neutrophils to the wound site (TGF-β-dependent)		
4	Rate of neutrophil apoptosis		
5	Rate of apoptotic neutrophil phagocytosis by pro-inflammatory macrophages		
6	Apoptotic neutrophil phagocytosis parameter		
7	Chemotactic migration of pro-inflammatory macrophages to the wound site (TGF-β-dependent)		
8	Rate of macrophage phenotype conversion		
9	Macrophage phenotype conversion parameter		
10	Rate of macrophage efflux by lymphatic system		
11	Rate of TGF-β production by pro-inflammatory macrophages		
12	Rate of TGF-β production by anti-inflammatory macrophages		
13	TGF-β degradation rate		
14	Rate of PDGF production by pro-inflammatory macrophages		
15	Rate of PDGF production by anti-inflammatory macrophages (10% of *k*_*PDGF_pro*_)		
16	PDGF degradation rate		
17	Rate of TNF-α production by pro-inflammatory macrophages		
18	Rate of TNF-α production by anti-inflammatory macrophages		
19	TNF-α degradation rate		
20	Rate of IL-1β production by pro-inflammatory macrophages		
21	Rate of IL-1β production by anti-inflammatory macrophages		
22	IL-1β degradation rate		
23	Rate of IL-6 production by pro-inflammatory macrophages		
24	Rate of IL-6 production by anti-inflammatory macrophages (10% of *k*_*IL6_pro*_)		
25	IL-6 degradation rate		
26	Rate of IL-10 production by pro-inflammatory macrophages		
27	Rate of IL-10 production by anti-inflammatory macrophages		
28	IL-10 degradation rate		
29	Rate of CXCL8 production by pro-inflammatory macrophages		
30	Rate of CXCL8 production by anti-inflammatory macrophages		
31	CXCL8 degradation rate		
32	Rate of IL-12 production by pro-inflammatory macrophages		
33	IL-12 degradation rate		
34	Rate of MIP-1α production by pro-inflammatory macrophages		
35	Rate of MIP-1α production by anti-inflammatory macrophages		
36	MIP-1α degradation rate		
37	Rate of MIP-2 production by pro-inflammatory macrophages		
38	Rate of MIP-2 production by anti-inflammatory macrophages		
39	MIP-2 degradation rate		
40	Rate of IP-10 production by pro-inflammatory macrophages		
41	Rate of IP-10 production by anti-inflammatory macrophages		
42	IP-10 degradation rate		
43	Rate of TNF-α production by active neutrophils		
44	Rate of IL-1β production by active neutrophils		
45	Rate of IL-6 production by active neutrophils		
46–48	Parameters of the feedback function *f* describing the inhibition of TNF-α by IL-10: $f=P_{46}e^{P_{47}IL10}+P_{48}$		
49–51	Parameters of the feedback function *f* describing the inhibition of IL-6 by IL-10: $f=P_{49}e^{P_{50}IL10}+P_{51}$		
52–54	Parameters of the feedback function *f* describing the inhibition of IL-1β by IL-10: $f=P_{52}e^{P_{53}IL10}+P_{54}$		
55–57	Parameters of the feedback function *f* describing the inhibition of TNF-α by TGF-β: $f=P_{55}e^{P_{56}TGF\beta }+P_{57}$		
58–60	Parameters of the feedback function *f* describing the inhibition of IL-1β by TGF-β: $f=P_{58}e^{P_{59}TGF\beta }+P_{60}$		
61–62	Parameters of the feedback function *f* describing the inhibition of IL-1β by IL-6:$f=\frac{P_{61}}{\left(P_{61}+IL6^{P_{62}}\right)}$		
63–64	Parameters of the feedback function *f* describing the inhibition of TNF-α by IL-6: $f=\frac{P_{63}}{\left(P_{63}+IL6^{P_{64}}\right)}$		
65–67	Parameters of the feedback function *f* describing the inhibition of IL-12 by TNF-α: $f=P_{65}e^{P_{66}TNF\alpha }+P_{67}$		
68	Parameter of the feedback function *f* describing the upregulation of IL-6 by TGF-β: $f=\frac{P_{68}TGF\beta }{\left(1+TGF\beta \right)}$		
69	Parameter of the feedback function *f* describing the upregulation of IL-10 by TGF-β: $f=\frac{P_{69}TGF\beta }{\left(1+P_{69}TGF\beta \right)}$		

The production rates are in units of ng∙cell^-1^∙h^-1^ and the degradation rates are in units of h^-1^. Parameters 46–69 are dimensionless parameters that describe positive and negative regulatory feedback functions for the production rates of certain molecular mediators. The form of the feedback functions was chosen by fitting different types of functions (e.g., linear, exponential, and polynomial) to experimental data and selecting the function that provided the best fit. The values of each parameter, along with the respective references to experimental studies from which each parameter was derived, are provided in Table S1 of [11].

The amount indices, Ψ_max_ and R_p_, for the majority of the model output variables were robustly regulated by the parameters representing the rate of macrophage influx (P#7) into the inflamed area and the rate of their efflux (P#10), respectively. Table 2 shows the first, second, and third most influential (based on the ranking of the corresponding sensitivities) model parameters for the inflammation indices for six specific model output variables. These variables (namely, TNF-α, IL-1β, IL-6, IL-10, total neutrophils, and total macrophages) are the ones that typically demonstrate abnormal characteristics during pathological inflammation [17, 34, 36, 42]. By definition, Ψ_max_ and R_p_ characterize the inflammation intensity at its peak and during its resolution (Fig 1). Therefore, these results are consistent with, and complement, our earlier findings identifying macrophage influx (P#7) and efflux (P#10) rates as the main regulators of kinetic trajectories during the initial and final phases of inflammation, respectively [11]. In addition to the well-pronounced regulation by macrophage flux rates, our local sensitivity analysis identified the TNF-α degradation rate (P#19) and the neutrophil influx rate (P#3) as robust regulators of the Ψ_max_ for the TNF-α and the neutrophil (N_tot_) model output variables, respectively (Table 2).

**Table 2:** Results of the model sensitivity analysis.

Sensitivity	Activation time (T_act_)					
	TNF-α	IL-1β	IL-6	IL-10	N_tot_	M_tot_
Highest	1 (2,774)	1 (2,724)	1 (3,039)	1 (2,470)	1 (4,035)	1 (4,681)
2^nd^ highest	13 (1,887)	13 (1,250)	13 (1,635)	13 (1,135)	13 (3,769)	13 (3,102)
3^rd^ highest	2 (839)	22 (624)	2 (785)	2 (610)	2 (371)	2 (371)
	**Peak height (Ψ**_**max**_ **)**					
	**TNF-α**	**IL-1β**	**IL-6**	**IL-10**	**N**_**tot**_	**M**_**tot**_
Highest	**19 (6,081**)	**22 (3,560**)	**7 (7,725**)	**7 (6,948**)	**3 (9,766**)	**7 (8,960**)
2^nd^ highest	17 (2,846)	7 (3,243)	23 (1,282)	28 (1,783)	1 (66)	1 (342)
3^rd^ highest	7 (404)	20 (1,842)	1 (284)	1 (355)	13 (35)	13 (233)
	**Resolution interval (R**_**i**_ **)**					
	**TNF-α**	**IL-1β**	**IL-6**	**IL-10**	**N**_**tot**_	**M**_**tot**_
Highest	10 (1,573)	10 (2,369)	1 (1,441)	10 (2,454)	1 (2,321)	10 (3,481)
2^nd^ highest	1 (1,146)	1 (913)	10 (1,009)	1 (984)	13 (1,412)	1 (1,025)
3^rd^ highest	7 (1,004)	13 (755)	13 (813)	13 (624)	7 (790)	13 (592)
	**Resolution plateau (R**_**p**_ **)**					
	**TNF-α**	**IL-1β**	**IL-6**	**IL-10**	**N**_**tot**_	**M**_**tot**_
Highest	**10 (8,753**)	**10 (9,205**)	**10 (8,994**)	**10 (9,724**)	**7 (2,203**)	**10 (9,805**)
2^nd^ highest	1 (284)	1 (162)	1 (221)	13 (32)	5 (2,092)	1 (23)
3^rd^ highest	7 (178)	13 (117)	13 (112)	1 (31)	10 (1,426)	13 (17)

In each cell, the entry without parentheses represents the assigned number (P#) of a particular model parameter, and the entry in parentheses represents the number of simulations (out of the total 10,000 simulations) for which the sensitivity of the indicated inflammation index (corresponding to the indicated model output variable) with respect to this parameter (see Eq 1) was the highest, 2^nd^ highest, and 3^rd^ highest across all 69 model parameters.

In contrast to the robust regulation of the amount indices, no model variables had their timing indices (i.e., T_act_ and R_i_) robustly regulated by a model parameter. Indeed, the largest sensitivity values for T_act_ and R_i_ corresponded to different parameters depending on the specific simulation (i.e., on the specific model parameter set). For all of the16 variables, the model parameter effecting the strongest T_act_ regulation in the largest fraction of the 10,000 inflammatory scenarios was the platelet degradation rate (P#1) (Table 2). Yet, this largest fraction (which we call the “robustness fraction”) was too small (~18–46%) to consider this regulation robust. For the R_i_, the parameter effecting the strongest regulation of this index in the largest fraction of scenarios was macrophage efflux rate (P#10) for 10 out of the 16 model output variables. However, the corresponding robustness fraction for it was even smaller (~15–35%). These results suggest that robust control of the timing indices may require a sophisticated strategy involving simultaneous modulation of multiple inflammatory mechanisms.

### Statistical Correlation between the Model Parameters and the Inflammation Indices

To gain additional insights into the regulation of the inflammation indices, we calculated the correlation coefficients (CCs) and the associated *p*-values between the inflammation indices of each model output variable and each model parameter (see Materials and Methods). The CC values ranged between −1 to +1 reflecting a negative or a positive index-parameter correlation, respectively. The signs of the CCs for the four indices are shown in S2 Fig. Absolute index–parameter correlations above 0.5 were considered *strong* [43, 44]. Among the correlations identified as strong, only correlations with *p* ≤ 0.05 were considered statistically significant.

**Fig 2:**
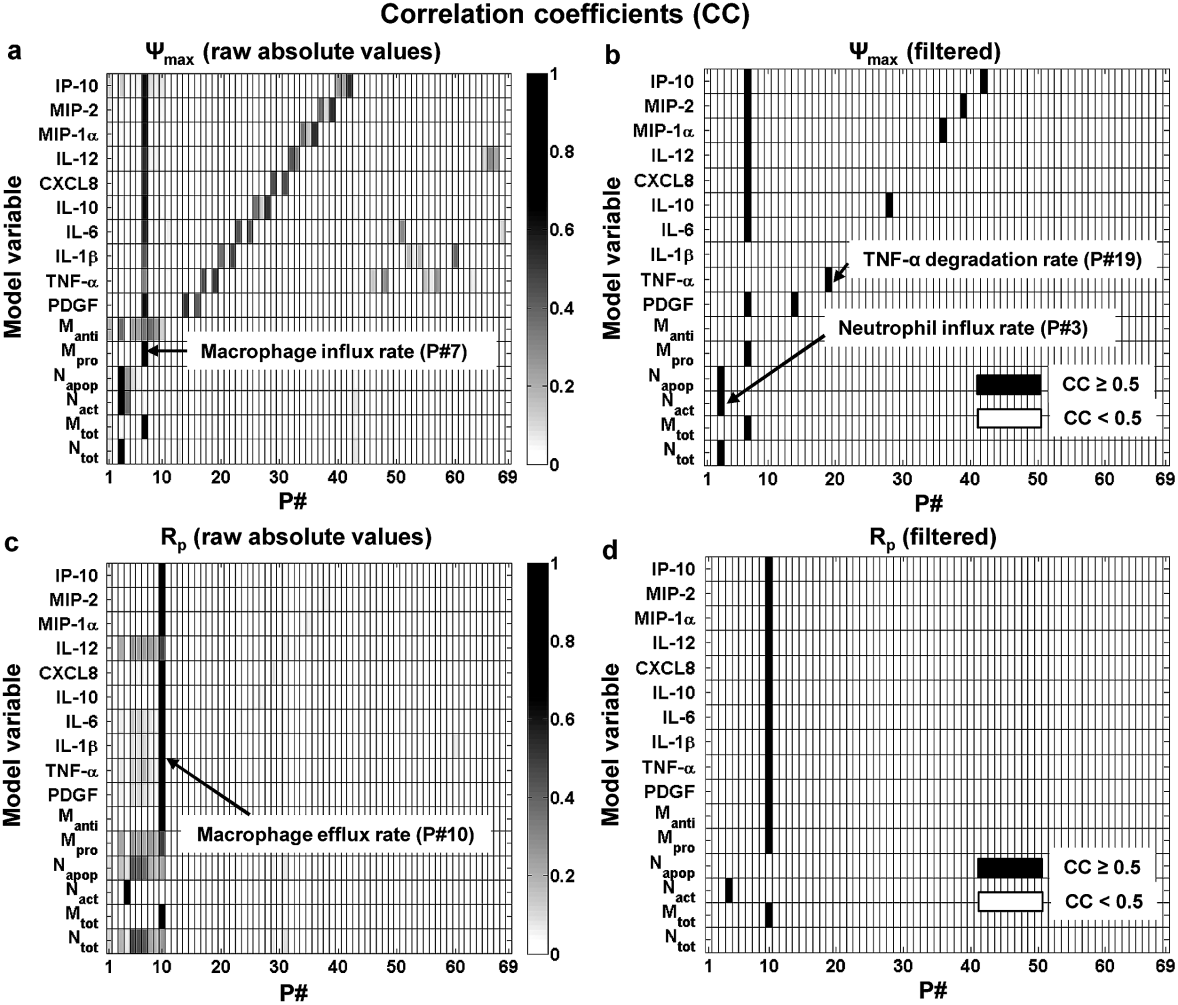
Correlation analysis for the model parameters (P#) and the amount indices of the model output variables. The shades of grey represent the absolute values of the correlation coefficients (CCs). Subplots **a** and **c** show raw absolute CC values for Ψ_max_ and R_p_, respectively. Subplots **b** and **d** show only the strong (i.e., CC ≥ 0.5) index-parameter associations for subplots **a** and **c**, respectively. See Table 1 for a list of all model variables and parameters.

For the amount indices, we detected a strong positive correlation between inflammation peak height (i.e., Ψ_max_) and macrophage influx rate (P#7) for 10 model output variables, including macrophages, IL-6, and IL-10 (Fig 2a and 2b). Furthermore, we found a strong positive correlation between the Ψ_max_ for neutrophils (N_tot_) and neutrophil influx rate (P#3) (Fig 2a and 2b), which was consistent with our sensitivity analysis results (Table 2). This result suggests that the inflammation indices for the neutrophil inflammatory response trajectory may depend on the degradation rates of CXCL8 (P#31) and TGF-β (P#13), which are the two main neutrophil chemoattractants in our model [11]. For the other amount index, i.e., R_p_, only one parameter [namely, macrophage efflux rate (P#10)] showed a strong (negative) correlation with a large number (namely, 13) of model output variables (Fig 2c and 2d). These findings were consistent with our sensitivity analysis results regarding the Ψ_max_ and R_p_ regulation by macrophage influx and efflux rates (Table 2).

**Fig 3:**
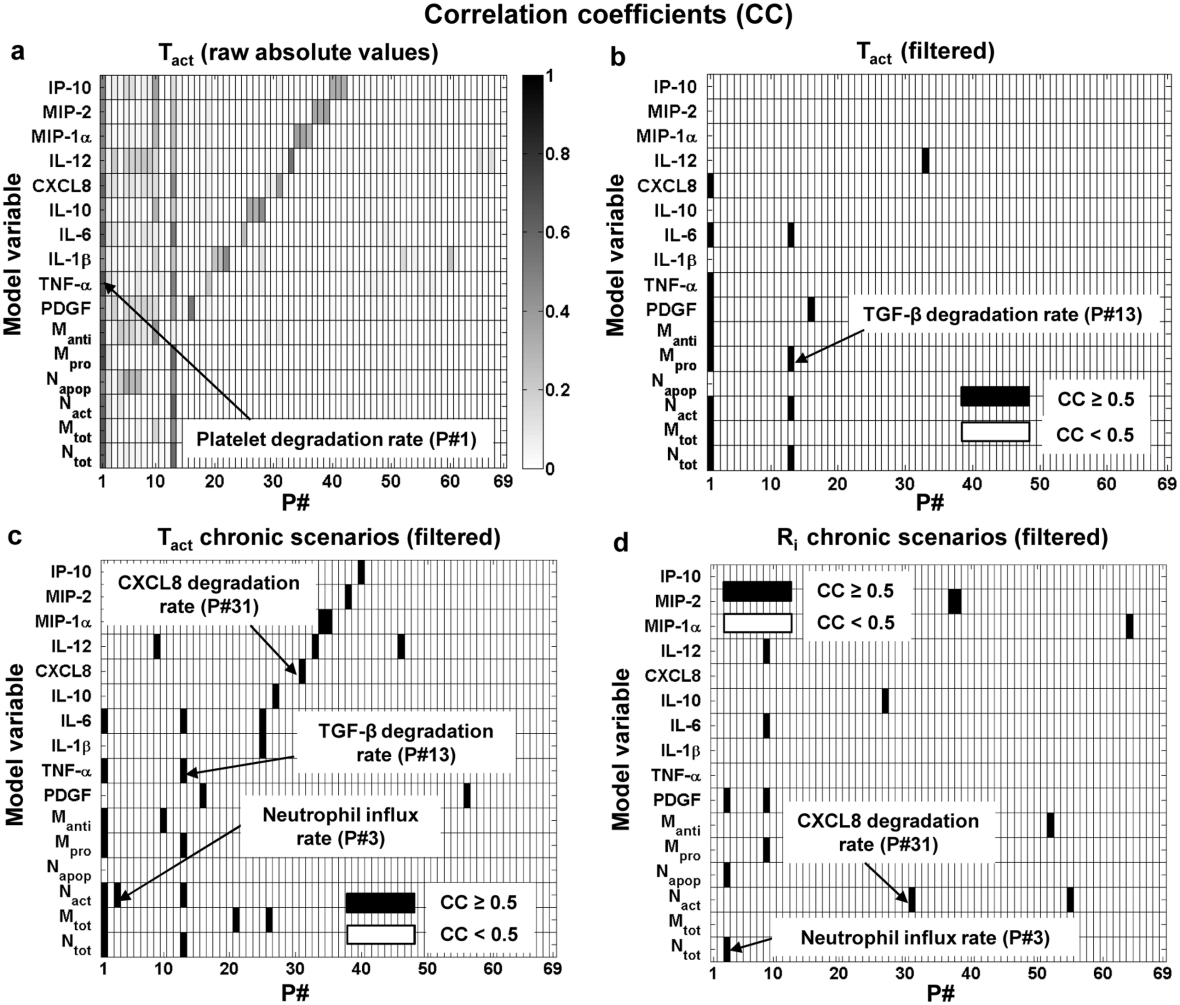
Correlation analysis for the model parameters (P#) and the timing indices of the model output variables. The shades of grey represent the absolute values of the CCs. Subplot **a** shows the raw absolute CC values for T_act_ calculated using the full set of 10,000 simulations. Subplots **b**, **c**, and **d** show only the strong (i.e., CC ≥ 0.5) index-parameter associations for T_act_ in the full set of 10,000 simulations, T_act_ in the “chronic” (see Materials and Methods) subset of simulations, and R_i_ in the “chronic” subset of simulations, respectively. See Table 1 for a list of all model variables and parameters.

For the timing indices, the correlation analysis highlighted a strong negative correlation between TGF-β degradation rate (P#13) and the T_act_ of four model variables, including neutrophils (N_tot_) and IL-6 (Fig 3a and 3b). Moreover, we detected a strong negative correlation between the platelet degradation rate (P#1) and inflammation activation time (i.e., the T_act_ index) for 9 model variables (Fig 3a and 3b). However, this relationship simply demonstrates the connection between T_act_ and the strength and persistence of inflammation-initiating stimuli, reflected in our model by the presence of platelets in the wound. Additionally, the R_i_ for the majority of the model variables, including macrophages and IL-6 (results not shown), exhibited a strong negative correlation with macrophage efflux rate (P#10), which could be expected based on our sensitivity analysis results (Table 2). Yet, our correlation analysis did not detect any other strong correlations for the timing indices.

The limited number of detected strong correlations prompted us to hypothesize that a larger number of strong correlations between the timing indices and the model parameters could be detected in a smaller set of simulations reflecting specific conditions, such as chronic inflammation. To test this, we divided the 10,000 simulations into two subsets, “acute” and “chronic” (see Materials and Methods). Then, for only the “chronic” subset, we performed the correlation analysis between each of the timing indices (i.e., T_act_ and R_i_) for the 16 model output variables and the 69 model parameters. As hypothesized, in this analysis many model parameters emerged as strongly correlated with T_act_ (18 parameters) and R_i_ (9 parameters), for different model variables (Fig 3c and 3d, respectively). In subsequent analyses, we specifically focused on TGF-β and CXCL8 degradation rates (P#13 and P#31, respectively), because they were strongly correlated with several key model outputs and those correlations were statistically significant. In summary, the correlation analysis provided us with candidate mechanisms for directly regulating both of the amount indices (via neutrophil influx rate and the macrophage flux rates) and the timing indices (via the TGF-β and CXCL8 degradation rates) of the model output variables.

The goal of model parameter randomization in our 10,000 simulations was to account for possible biological variability in inflammation scenarios [11]. While wider variation ranges for the parameters may allow for a fuller representation of this variability, such random, simultaneous, uncorrelated variations may also introduce excess noise that could mask the biologically relevant patterns we want to detect. An informative analysis should therefore utilize parameter ranges sufficiently wide to represent variation and yet narrow enough to define the vicinity of our carefully chosen default parameter set, which had been derived directly from experimental data and represented the expected “typical” injury-triggered inflammation scenario [11]. To assess the impact of large parameter deviations, we performed the sensitivity and correlation analysis for an additional set of 40,000 simulations. In these simulations, the parameters were randomly and uniformly sampled from a 9-fold range (i.e., 3-fold down and 3-fold up) around the default parameter values. In the sensitivity analysis, the introduction of these larger parameter deviations reduced by 5–30% (results not shown) the robustness of the Ψ_max_ regulation by macrophage influx rate for the six different outputs shown in Table 2. Similarly, the robustness of the R_p_ regulation by macrophage efflux rate was reduced by 1–14% (results not shown) in comparison with the results shown in Table 2. However, in the correlation analysis, the major trends [i.e., the strong correlation between the Ψ_max_ and macrophage influx rate and the strong correlation between the R_p_ and macrophage efflux rate] were preserved, while other strong correlations (Figs 2 and 3) were not detected in the case of large parameter deviations (S3 Fig). Thus, it appears that some of the detected biological patterns are particularly strong in the fewfold vicinity of our default parameter set, which reflects their dependence on specific type of inflammatory scenario.

### Partial Restoration of the Normal Values of the Inflammation Indices in Chronic Inflammation by Model Parameter Modulation

**Fig 4:**
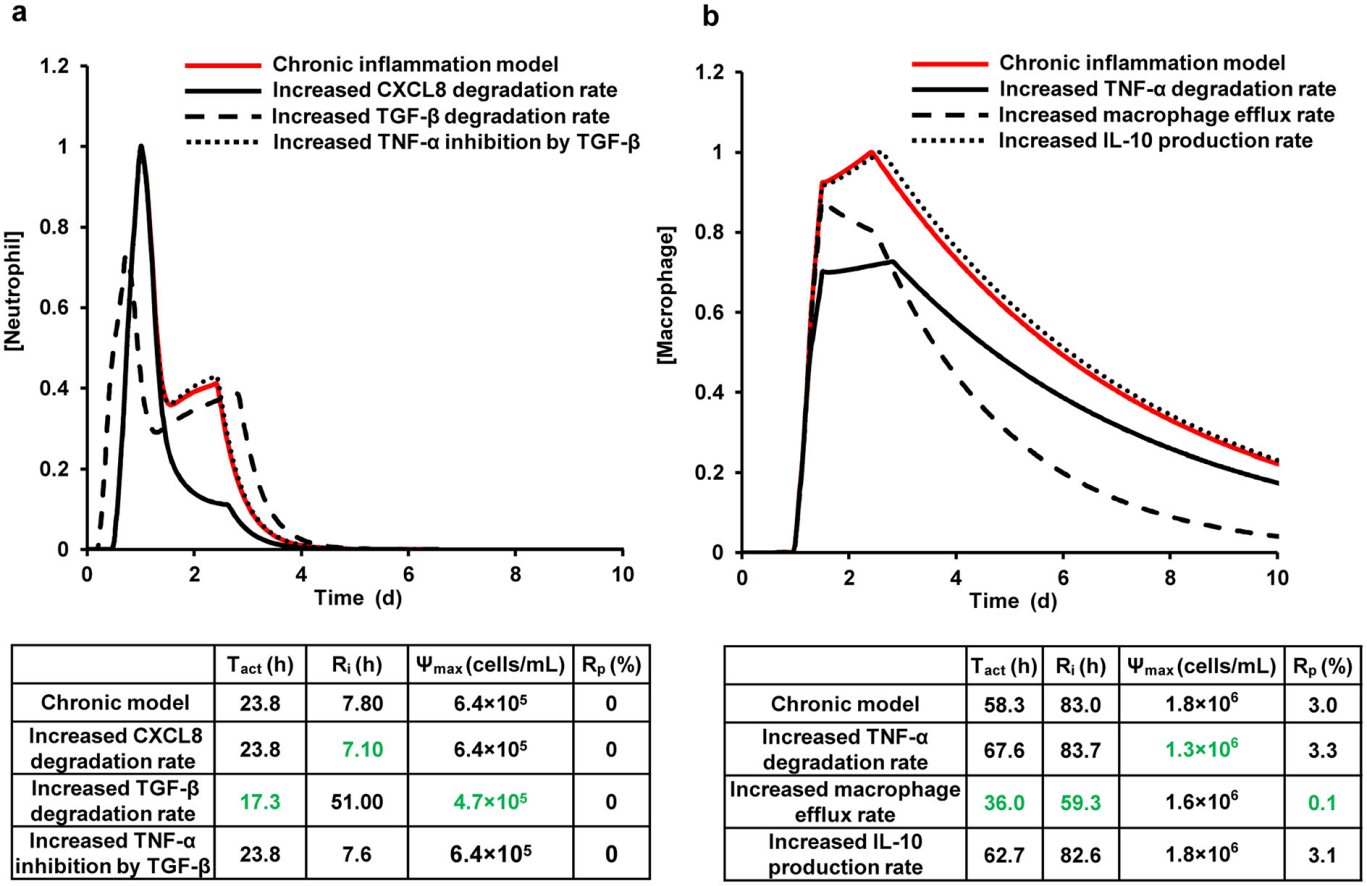
Inflammation index regulation by modifying the model parameters identified in the sensitivity and correlation analyses. Shown are the normalized total neutrophil (**a**) and total macrophage (**b**) concentrations during chronic (solid red) inflammation. We simulated chronic inflammation by increasing the macrophage influx rate parameter by 5 fold, a strategy we employed in our previous model [11]. These curves appeared in our previous model results (see Figure 5 in [11]) and are reproduced here for comparison purposes. Neutrophil restoration modifications included increasing the CXCL8 degradation rate by 5 fold (solid black), increasing the TGF-β degradation rate by 15 fold (dashed black), and increasing TNF-α inhibition by TGF-β by 5 fold (dotted black). Macrophage restoration modifications comprised increasing the TNF-α degradation rate by 20 fold (black solid), increasing the macrophage efflux rate by 2 fold (dashed black), and increasing IL-10 production rate by 5 fold (dotted black). These parameter-specific fold changes were chosen so as to reduce the respective parameter-specific target inflammation indices by at least ~10% during a chronic inflammatory scenario (the target indices are defined in the description of the six parameter modification strategies in the Results Section). All the model-predicted values were normalized to the respective maximum values from the chronic inflammation simulations. The tables in the figure show the quantitative index values calculated from the respective simulated kinetic trajectories.

The results of our sensitivity and correlation analyses (Table 2 and Figs 2 and 3) suggested that specific inflammation indices of the model variables can be considerably impacted by the following five model parameters: macrophage influx and efflux rates and the TNF-α, CXCL8, and TGF-β degradation rates. We therefore wanted to investigate whether modulation of these parameters during chronic inflammation could result in (at least, partial) restoration of the normal (i.e., acute-inflammation) values of the respective inflammation indices. We addressed this question for all of the five parameters except macrophage influx rate, because we used its modulation to *induce* chronic inflammation in the model (see caption for Fig 4). Using the same protocol as implemented in our previous modeling study (see Figure 5 in [11]), we simulated a chronic inflammatory scenario (Fig 4a and 4b, red line). Here, we specifically focused on the kinetic trajectories for neutrophils and macrophages, because their kinetic behavior is well studied and is known to be disrupted during delayed (or chronic) inflammation [34, 45].

To investigate the regulation of neutrophil and macrophage inflammatory trajectories, we repeated the chronic inflammation simulations upon modifying the values of the parameters mentioned above, as follows. In the chronic inflammation simulation for neutrophil regulation, we implemented two distinct parameter modification strategies. In one strategy, we increased the CXCL8 degradation rate, which caused the R_i_ for the neutrophils to decrease (Fig 4a, solid black line and green values in the table) compared to the chronic scenario with no modification (Fig 4a, red line). In the other strategy, we increased the TGF-β degradation rate, which caused both T_act_ and Ψ_max_ for the neutrophils to decrease (Fig 4a, dashed black line, green values in the table) compared to the chronic scenario with no modification (Fig 4a, red line).

Similarly, for macrophage regulation, we introduced two distinct parameter modification strategies into the chronic inflammation simulation. In the first strategy, we increased the TNF-α degradation rate, which caused the Ψ_max_ for the macrophage variable to decrease (Fig 4b, solid black line and green values in the table) compared to the chronic scenario with no modification (Fig 4b, red line). In the second strategy, we increased the macrophage efflux rate, which caused the T_act_, R_i_, and R_p_ for the macrophage variable to decrease (Fig 4b, dashed black line and green values in the table) compared to the chronic scenario with no modification (Fig 4b, red line). The observed decrease in the inflammation index values suggested that the considered parameter modifications can partially *restore* acute inflammatory kinetics, i.e., can bring the inflammation index values for a chronic inflammatory scenario closer to those for an acute inflammatory scenario.

The analyses described above were supplemented with modulation of other parameters. This was done to illustrate that not all model parameters exhibiting strong correlations with certain model variables could effectively regulate their indices. For example, the IL-10 production rate parameter (P#26) was strongly and negatively correlated with the macrophage T_act_ (Fig 3c), and the parameter for TNF-α inhibition by TGF-β (P#55) had a strong and negative correlation with the neutrophil (N_tot_) R_i_ (Fig 3d). However, increasing those two parameters did not result in a significant modulation of the respective neutrophil or macrophage indices (Fig 4a and 4b, dotted lines). The model parameters that effected weak inflammatory index regulation or exhibited low-confidence correlations with the inflammatory indices were not selected for further analysis.

### Regulation of the Inflammation Indices by Inflammatory Mediator Inhibition: Modeling Predictions and Validation

**Fig 5:**
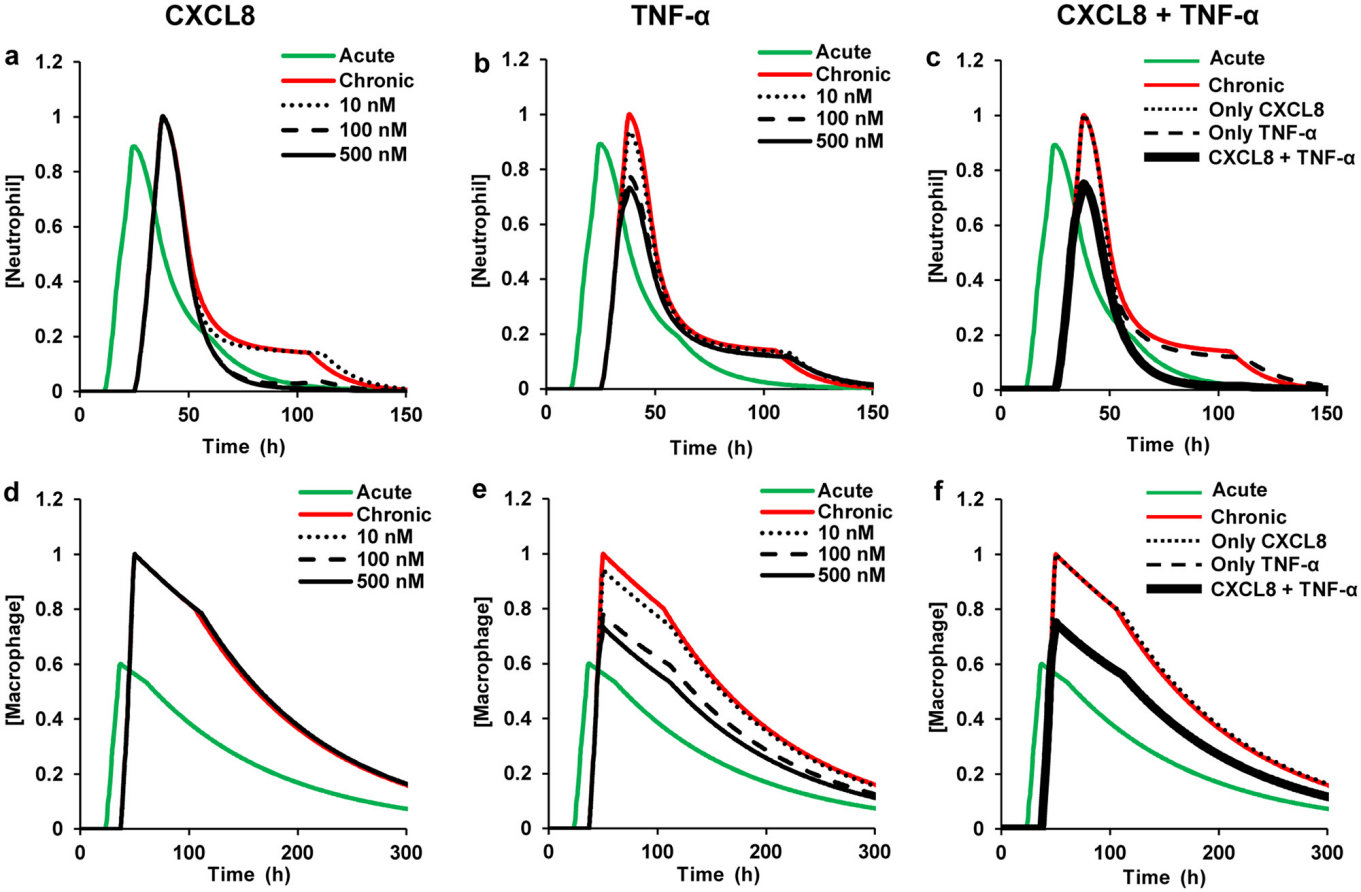
Total neutrophil and total macrophage concentrations for inflammatory mediator inhibition by different inhibitor concentrations. Green and red lines represent model predictions for acute and chronic inflammation, respectively. We simulated chronic inflammation by increasing the initial platelet concentration by 100 fold, increasing the macrophage influx rate parameter by 1.5 fold, and simultaneously decreasing the platelet degradation rate parameter by 1.5 fold. We chose to increase the macrophage flux rate and decrease the platelet degradation rate because these rates were identified as robust regulators of the Ψ_max_ and T_act_ indices for many inflammatory cellular and molecular components, as seen in our sensitivity and correlation analysis results (Table 2 and Figs 2 and 3). Moreover, the role of macrophage influx rate as a chronic inflammation driver has been shown in our previous computational analysis [11]. Experimental studies indicate that a relatively higher inflammation-inducing stimulus often results in chronic inflammation [17, 34]. Thus, we chose to modify the initial platelet concentration, which reflects injury severity in our model. The fold increase/decrease in these parameters was chosen based on the ability of the parametric changes to simulate the experimentally determined inflammatory trajectories from [34]. Black lines represent model predictions for different inhibitor concentrations for the respective mediators. Each inhibitor concentration was added at the time of inflammation initiation (*t* = 0) during chronic inflammation. **a** and **d**: Normalized neutrophil and macrophage concentrations during CXCL8 inhibition for inhibitor concentrations of 10 nM (dotted black), 100 nM (dashed black), and 500 nM (solid black). **b** and **e**: Normalized neutrophil and macrophage concentrations during TNF-α inhibition for inhibitor concentrations of 10 nM (dotted black), 100 nM (dashed black), and 500 nM (solid black). **c** and **f**: Normalized neutrophil and macrophage concentration during combined inhibition of both CXCL8 and TNF-α, with each inhibitor concentration set to 200 nM (solid black). Also shown are the normalized neutrophil and macrophage concentrations for individual inhibition of CXCL8 (dotted black) and TNF-α (dashed black), with the respective inhibitor concentrations equal to 200 nM. All model-predicted values were normalized to the respective maximum values from the chronic inflammation simulations.

**Fig 6:**
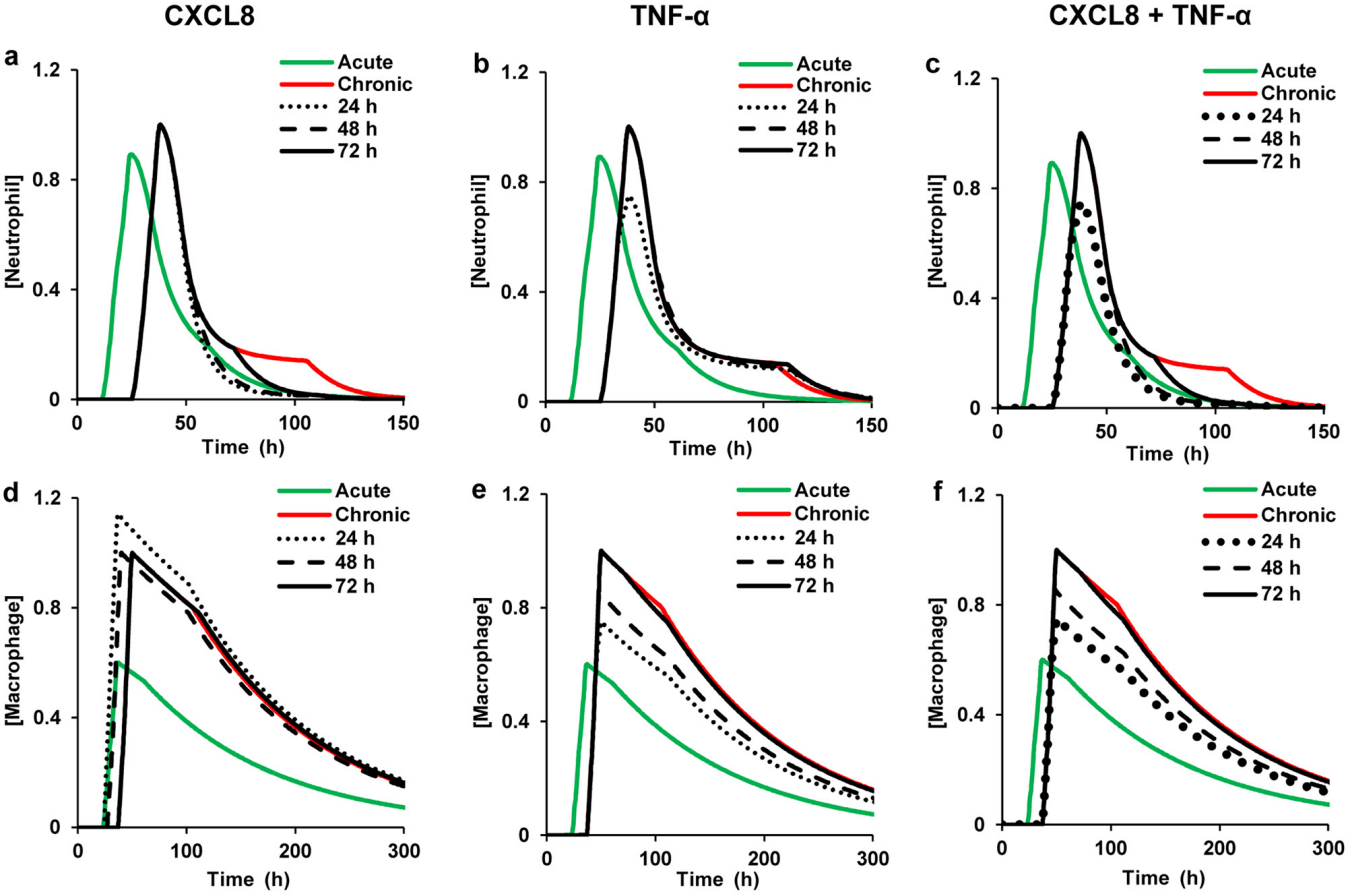
Total neutrophil and total macrophage concentrations for inflammatory mediator inhibition at different time points. Green and red lines represent model predictions for acute and chronic inflammation, respectively. We simulated chronic inflammation by increasing the initial platelet concentration by 100 fold, increasing the macrophage influx rate parameter by 1.5 fold, and simultaneously decreasing the platelet degradation rate parameter by 1.5 fold. In all the subplots, the time points of inhibitor addition (represented by the black lines) are as follows: dotted, 24 h; dashed, 48 h; and solid, 72 h. Shown are normalized neutrophil (**a**-**c**) and normalized macrophage (**d**-**f**) kinetics for CXCL8, TNF-α, and combined CXCL8 + TNF-α inhibition. The concentration of each of the considered inhibitors was equal to 200 nM. All model-predicted values were normalized to the respective maximum values from the chronic inflammation simulations. In our simulations, the 72 h time point was chosen for mediator inhibitor addition based on an experimental study that evaluated the efficacy of several pro-resolution drugs in a mouse peritoneal infection model [17]. Furthermore, we chose the 24 h and 48 h time points for mediator inhibitor addition because they represent the times at which the neutrophil and macrophage concentrations peak, respectively, in our acute inflammation simulation [11].

Because the TGF-β, CXCL8, and TNF-α degradation rates represent inherent biochemical properties of the respective molecular mediators, they cannot be easily changed *in vivo*. We therefore wanted to test whether a mechanistically distinct process, such as cytokine inhibition, could be used to obtain functionally similar outcomes. For this purpose, we extended our model [11] to include the kinetics of inhibitors for TGF-β, CXCL8, and TNF-α and performed inhibition kinetics simulations for chronic inflammatory scenarios (Figs 5 and 6 and S1). We derived the values for the association (*k*_*on*_) and dissociation (*k*_*off*_) rate constants (Eq 2, defined in the Materials and Methods Section) for each mediator inhibitor from literature data (S1 Table). In our simulations, CXCL8 inhibition primarily regulated inflammation timing (specifically, the R_i_ index for the neutrophil variable; Figs 5a and 6a, black lines), whereas TNF-α and TGF-β inhibition primarily regulated inflammation intensity (specifically, the Ψ_max_ and R_p_ indices for both neutrophil and macrophage variables) (Figs 5b and 5e and 6b and 6e; S1a and S1d Figs, black lines).

For each inhibitor, we performed simulations for three different inhibitor concentrations. We used inhibitor concentrations of 10 nM, 100 nM, and 500 nM for both the TNF-α and CXCL8 inhibitors. For the TGF-β inhibitor, we used the concentrations of 1 nM, 20 nM, and 200 nM. We found that the inhibitors for different targets are most effective within concentration ranges that can be vastly different (Figs 5 and S1). Indeed, the CXCL8 and TNF-α inhibitors were most effective in restoring (at least, partially) their target indices when the inhibitors were added at concentrations ≥100 nM (Fig 5a, 5b and 5e), while TGF-β inhibition was effective for inhibitor concentrations in the range ~1–10 nM (S1 Fig). These results attest to the efficacy of the simple strategy involving the inhibitor addition at time 0.

To validate our modeling predictions regarding TNF-α inhibition, we compared our simulations with the experimental data generated for H1N1 virus-induced lung inflammation regulated by the TNF-α inhibitor etanercept [46]. These data characterized three scenarios: *1*) control (i.e., no infection and, therefore, no inflammation), *2*) inflammation with infection without added TNF-α inhibitor, and *3*) inflammation with infection and with added 200 nM TNF-α inhibitor. From these data, we calculated the ratios of the total neutrophil and macrophage concentrations for the inflammation scenario without the TNF-α inhibitor to the corresponding concentrations for the inflammation scenario with added TNF-α inhibitor. We compared these ratios with the respective ratios calculated from our model simulation of injury-induced chronic inflammation. There was a reasonable agreement between the simulation-derived and experiment-derived ratios (Table 3). Note that, in our analysis, we did not attempt to model the nonzero neutrophil and macrophage concentrations detected for the experimental control scenarios, because in our model, which represents *extravascular* space, the concentrations of neutrophils and macrophages in the absence of inflammation are zero. In contrast, the control experiments measured the cell concentrations in entire lung lobes, which included cells present in the vasculature, resulting in nonzero neutrophil and macrophage concentrations even in the absence of inflammation. We chose this particular experimental study for the validation because TNF-α is a key pro-inflammatory cytokine that is secreted by inflammatory cells in most inflammatory scenarios, and whose expression and regulatory functions are largely independent of the specific inflammation-inducing stimuli (e.g., viral load, LPS [47], and wounding [39]). Thus, despite the difference in the inflammation-inducing stimulus between the experimental study and our modeling study (viral loading in lungs vs. injury, respectively), the reasonable agreement of the neutrophil and macrophage kinetics between the two studies suggests that our model adequately captured TNF-α inhibition.

**Table 3:** Experimentally observed and model-simulated total neutrophil and total macrophage concentration ratios for day 2 and day 4 of the inflammatory response.

Day	Experimental data	Model simulation
	**Total neutrophils**	
Day 2	2.1	1.3
Day 4	1.4	1.2
	**Total macrophages**	
Day 2	1.3	1.3
Day 4	1.3	1.4

Numbers in the table represent the ratio of the respective inflammatory cell concentration for the inflammation scenario without TNF-α inhibitor to the corresponding concentration for the inflammation scenario with TNF-α inhibitor added.

To test whether the timing of inhibitor administration can impact the index restoration outcomes, we performed simulations in which the inhibitors were added at three different time points (i.e., 24, 48, and 72 h) during chronic inflammation. We chose 24 h as the earliest intervention time point because neutrophils are the first blood leukocytes to arrive at the inflammation site, and 24 h approximately corresponds to the neutrophil peak time for acute inflammatory response [35, 48]. Macrophages peak at 48 h, which motivated our choice of the second intervention time. For all the simulations, the added CXCL8 and TNF-α inhibitor concentrations equaled 200 nM (selected based on the observed effective range >100 nM, Fig 5a, 5b and 5e). In the case of CXCL8, the inhibitor added at 24 h was more effective in reducing the R_i_ for the neutrophil variable than the inhibitor added at other time points (Fig 6a, dotted black line). For TNF-α, the inhibitor added at 24 h provided a more complete restoration of the Ψ_max_ for both neutrophils and macrophages to its acute-inflammation value than the TNF-α inhibitor added at later time points (Fig 6b and 6e, dotted black lines).

A nearly identical effect on the neutrophil R_i_ and macrophage Ψ_max_ was observed when the CXCL8 and TNF-α inhibitors, respectively, were added at 48 h (Fig 6a and 6e, dashed black lines nearly overlapped the dotted black lines). Mediator addition at 72 h showed the least degree of restoration in the neutrophil R_i_ and macrophage Ψ_max_ among the three inhibitor addition times analyzed (Fig 6a and 6e, black solid lines). However, this observed effect of mediator inhibition on the inflammation indices was not monotonic. Specifically, when the CXCL8 and TNF-α inhibitors were added at 36 h (results not shown), the degree of restoration in the neutrophil R_i_ and macrophage Ψ_max_ values, respectively, was less than that when the mediator inhibitors were added at the 48 h time point. Experimental data from cytokine inhibition studies [49, 50] support the possibility of this type of non-monotonic behavior, which may be due to the complex nonlinear functional dependencies at work in the system. In summary, the CXCL8 and TNF-α inhibitors were characterized by similar optimal-efficiency concentration ranges and preferred administration timing regimens, but distinct preferentially regulated inflammation indices. Among the three time points considered, mediator inhibition was most effective when introduced during peak neutrophil response (i.e., at 24 h). For TGF-β, however, the inhibition outcomes were more complicated (see S1 Fig and S1 Text).

Because the neutrophil is the primary microbicidal cell type that produces powerful cytotoxic molecules that can potentially destroy the surrounding healthy tissue, (at least partial) restoration of the “normal” (i.e., acute-inflammation) timing and intensity of the neutrophil surge is essential for the resolution of chronic inflammation [51]. Based on the preferential regulatory action of individual CXCL8 and TNF-α inhibitors on the total neutrophil R_i_ and Ψ_max_, respectively (Fig 5a and 5b), we hypothesized that simultaneous inhibition of these two mediators in chronic inflammation might induce simultaneous restoration of the normal values for both of these indices. We tested this hypothesis by simulating the effect of adding both CXCL8 and TNF-α inhibitors at time 0. Each of the two inhibitors was added at a concentration of 200 nM. As hypothesized, the combined inhibition resulted in simultaneous restoration of both the R_i_ and Ψ_max_ of the total neutrophil trajectory in a chronic inflammation simulation (Fig 5c, solid black line). Similarly to the action of the CXCL8 inhibitor (Fig 6a), the combined inhibition of CXCL8 and TNF-α was most effective at 24 h (Fig 6c and 6f, dotted black lines). These results suggest that “cocktails” of therapeutic agents with different targets may provide strategies of improved efficacy for simultaneous control of both timing and intensity of inflammation after wounding.

## Discussion

Timely resolution of inflammation following an injury, infection, or disease is essential for the maintenance of healthy tissue [2, 52]. Despite ongoing research and development efforts, current anti-inflammatory therapies are only modestly effective and have significant negative side effects [9, 53, 54]. This work was motivated by the need to identify molecular mechanisms that could serve as targets for intervention strategies intended to modulate chronic inflammatory responses. Chronic inflammation may have distinct phenotypic manifestations depending on the inflammatory condition, e.g., increased apoptotic neutrophil levels in diabetic ulcers [36], or increased levels and prolonged presence of classically activated macrophages in ischemic wounds [45], or prolonged oscillations in the levels of inflammatory cells and cytokines [55]. Here, we focused on chronic inflammation characterized by heightened levels and/or delayed resolution timing for kinetic trajectories describing accumulation and depletion of the inflammatory cell types and molecular mediators in comparison with acute inflammation [2]. In our simulations (both acute and chronic), the kinetic trajectories of all the inflammatory components return to their baseline levels following a peak triggered by inflammation initiation. We used our computational model of injury-initiated local inflammatory response [11] to identify the mechanistic factors regulating the four inflammation indices, i.e., Ψ_max_, R_p_, T_act_, and R_i_ (Fig 1), which were recently introduced as quantitative markers of the levels and timing of the inflammation time course [1, 3, 13, 17].

Our sensitivity analysis elucidated the effects of parameter changes on the inflammation indices. For the majority of the model output variables, the amount indices (i.e., Ψ_max_ and R_p_) were robustly regulated by macrophage influx (P#7) and efflux (P#10) rates, respectively, in the 10,000 performed simulations (Table 2). Because macrophages are major cytokine-producing cells at the site of inflammation [39], identification of the macrophage flux rates as critical inflammation intensity control mechanisms is, perhaps, not surprising. However, the kinetics of each cytokine are additionally affected by a large number of factors, such as its individual production and degradation rates, feedback parameters, cell phenotype conversion rates, etc. In view of this diversity, identification of the macrophage flux rates as the *only* robust modulators for almost all model variables is an unexpected result. Interestingly, these results are supported by experimental studies involving direct macrophage manipulation [36, 37, 39, 40]. For example, elevated macrophage abundance was shown to cause wound fibrosis [40] and delayed wound healing [38]. Moreover, reducing the levels of functional macrophages in wounds can severely delay the wound healing time in mouse models [36, 37, 39].

The macrophage flux rates, being strong regulators of the intensity inflammation indices, (Table 2 and Fig 2), are in fact the primary targets of certain recently emerged pro-resolution molecular mediators, such as lipoxins and resolvins [4]. By perturbing the recruitment mechanisms of neutrophils and macrophages in our model (Fig 4), we were able to reproduce the experimentally observed effects of exogenous delivery of resolvins and lipoxins in models of murine peritonitis (Fig 4, black lines and green values in the tables), e.g., a reduction in the total neutrophil Ψ_max_ and T_act_ (see Table II in [1]), and a reduction in the total macrophage Ψ_max_ (see Figure 5 in [17]). While these experiments addressed only a handful of specific inflammatory scenarios, the robustness of the inflammation index regulation by the macrophage flux parameters, detected in our sensitivity analysis (Table 2), attests to the general nature of this regulation type. It should be noted, however, that the macrophage influx rate parameter (P#7) in our model is a lumped parameter representing macrophage chemotaxis stimulated by any combination of four distinct macrophage chemoattractants (specifically, TGF-β, PDGF, TNF-α, and MIP-1α) (see Table 1 in [11]). Likewise, the neutrophil influx rate parameter (P#3), which robustly regulates the total neutrophil Ψ_max_ (Table 2), characterizes the chemotaxis of active neutrophils stimulated by any combination of TGF-β and CXCL8. Thus, the sensitivity analysis alone [which identified P#7 and P#3 as influential parameters (Table 2)] was not sufficient for obtaining deeper insights into the specific mechanistic factors regulating the macrophage and neutrophil influx rates. This was accomplished in our parameter/output correlation analysis.

The correlation analysis explored the effects of simultaneous, random parameter variations within specified limits. The analysis identified a number of functional associations between model parameters and inflammation indices (Figs 2 and 3). Consistently with the sensitivity analysis results, macrophage influx and efflux rates were strongly correlated with Ψ_max_ and R_p_ of model outputs (Fig 2). However, in contrast to the sensitivity analysis results, where the timing indices (i.e., T_act_ and R_i_) did not display robust regulation by any of the kinetic model parameters across the 10,000 simulations, a strong negative correlation was observed between the platelet degradation rate and the T_act_ of the majority of output variables (Fig 3a and 3b). This correlation may simply reflect that the presence of platelets in the wound area is the only inflammation-initiating stimulus in our model. While other mediator-releasing cells (e.g., endothelial cells, resident macrophages, mast cells, etc. [56]) may contribute to the inflammatory response, this simplifying modeling assumption is in accord with the prominent roles played by platelets in initiating wound healing [57, 58]. Of the many platelet-secreted molecular mediators facilitating inflammation initiation [57], we only modeled the most potent neutrophil and macrophage chemoattractant, i.e., TGF-β [59, 60]. The results of this choice are evident from the strong correlations between the TGF-β degradation rate and key inflammatory components, such as total neutrophils, pro-inflammatory macrophages, and IL-6 (Fig 3b).

Overall, R_i_ was the least sensitive of all the inflammation indices and had a limited response to the parameter variations applied, which was consistent with its experimentally detected insensitivity [1]. Notably, for a subset of simulated scenarios reflecting chronic inflammation, certain parameters (e.g., TGF-β and CXCL8 degradation rates) exhibited a strong correlation with the T_act_ and R_i_ values for many model output variables, including key outputs such as total neutrophils, TNF-α, and IL-6 (Fig 3c and 3d). Thus, our correlation analysis identified relationships between specific inflammation indices and the specific molecular components regulating the macrophage (TGF-β, Fig 3c) and neutrophil (TGF-β and CXCL8, Fig 3c and 3d) influx.

We extended our model to describe inflammatory mediator inhibition kinetics in order to mechanistically represent the regulatory effects of inhibiting TNF-α, CXCL8, and TGF-β (Figs 5 and 6; S1 Fig, black lines). Mediator inhibition could provide a pharmacologically feasible strategy to mimic the functional effects of the modulation of the neutrophil and macrophage flux mechanisms, as well as of the modulation of TNF-α, CXCL8, and TGF-β degradation rates, which we identified as robust regulators of specific inflammatory indices. In our simulations, individual and combined mediator inhibition demonstrated the potential for significantly improved restoration of acute-scenario kinetics for specific cell types in certain injury-initiated chronic inflammatory scenarios (Figs 5 and 6). Interestingly, the molecular mediators TGF-β and CXCL8, which were identified as strong regulators of the timing indices T_act_ and R_i_ (Fig 3), successfully regulated the amount indices Ψ_max_ and R_p_ of neutrophils and macrophages (Figs 5 and S1). This suggests that CXCL8 and TGF-β may be the primary contributors to the neutrophil and macrophage abundance in the wound, respectively. Yet, the detected neutrophil Ψ_max_ regulation by TNF-α inhibition (Fig 5) was surprising because, unlike CXCL8, TNF-α is not a chemoattractant for neutrophils. TNF-α has been reported to be pro-apoptotic to neutrophils in specific concentration ranges [61]. It is conceivable that the non-linear feedback effects present in our model allowed us to capture this indirect effect of the neutrophil Ψ_max_ regulation by TNF-α.

The detected difference in regulation robustness between the timing and amount inflammation indices appears to be an intrinsic property of the inflammation regulation system. Interestingly, a regulation dichotomy between timing and amount properties of the output kinetics has been detected for other systems in molecular biology. For example, in bacterial signal transduction systems, the amount characteristics of the response curves are primarily defined by the numerical values of the systems’ kinetic parameters, whereas the response timing properties are determined largely by the systems’ architecture and are less sensitive to parameter variation [14]. Therefore, a lack of robust patterns characterizing the control of response timing via kinetic parameter modulation might be a common property of biological control systems.

Traditional anti-inflammatory therapies, such as nonsteroidal anti-inflammatory drugs targeting the production of prostaglandins [10], have focused on the inhibition of pro-inflammatory pathways. More recently, administration of pro-resolution molecular agents, such as lipoxins and resolvins, has shown promise in promoting inflammation resolution and is now a topic of active research [4, 62]. Direct inhibition of inflammatory mediators is another therapeutic strategy that has seen moderate success for specific inflammatory conditions. For example, TNF-α inhibition and IL-1β inhibition have been successful as therapies for rheumatoid arthritis (RA) [8, 9] and chronic inflammatory pathologies associated with cancer [63], respectively. Previously published reports suggest that single mediator inhibition strategies are useful predominantly in chronic inflammation scenarios driven by specific mediators and/or when only one inflammation system component is affected, e.g., in the neutrophil overload condition [36]. Our results are consistent with these findings and suggest that the observed therapeutic effects can be explained by preferential regulation of a given inflammation index by a specific mediator. Indeed, TGF-β and TNF-α inhibition regulate the total neutrophil and total macrophage T_act_ and Ψ_max_ (Figs 4a and 4b and 5b and 5e and 6b and 6e and S1a and S1d), while CXCL8 inhibition generally regulates the total neutrophil R_i_ (Figs 5a and 6a).

The generality of our approach suggests that TNF-α inhibition could potentially be used (alone or in combination with other mediator inhibitors) for a variety of inflammatory conditions characterized by elevated neutrophil and macrophage levels, such as traumatic injuries [64, 65]. TGF-β regulation has largely been studied for its role in fibrosis during the later stages of wound healing [66, 67]. Our results show that TGF-β inhibition might have a role in promoting the resolution of the crucial *inflammatory* stage of wound healing. In fact, recent clinical trials have begun evaluating the efficacy of TGF-β inhibition for various inflammatory pathologies, such as cancer [68]. CXCL8 inhibition has until now been speculated to have a role in inflammatory airway diseases, such as chronic obstructive pulmonary disease [69] and severe asthma [53]. Our results indicate a potential role for CXCL8 inhibition in timely neutrophil resolution during injury-initiated inflammation that warrants further experimental investigation. For pathological situations affecting several components of the inflammation process, the use of combination mediator therapy has been recently proposed and is being tested in clinical trials for rheumatoid arthritis [54]. Encouragingly, our simulations showed that a combined intervention targeting both TNF-α and CXCL8 in a chronic inflammatory scenario reduced both Ψ_max_ and R_i_ for the total neutrophil concentration and thereby restored the near-“normal” (in comparison with the acute inflammatory scenario) total neutrophil kinetic trajectory (Figs 5c and 6c). Overall, our modeling results suggest that individual and combined mediator inhibition approaches may be applicable during *trauma-induced* chronic inflammation, in addition to the inflammatory conditions for which they are currently indicated or being tested. Our model may be used to test the efficacy of different intervention strategies, involving combined administration of inhibitors for TNF-α, TGF-β, and CXCL8, and to possibly determine the intervention timing. The use of robust control methodologies with our model might facilitate the design of improved parameter selection strategies to simulate chronic inflammation and to optimize drug administration regimens [70].

The main limitations of our approach arise from the computational nature of our study and from the assumptions made during the computational model development [11]. First, in our sensitivity analysis, we only examined the effects of parameter variations in the vicinity of the default parameter set. Larger (e.g., several‐fold) parameter changes, which could represent a higher degree of dysregulation or severity of inflammation, were not examined. Nevertheless, local sensitivity analysis is an established research methodology that allowed us to generate model results consistent with experimental data (Table 2) [11, 19, 71–74]. While this methodology is not designed to investigate simultaneous variations in several parameters, simultaneous parameter variation was a part of our correlation analysis strategy. Moreover, the effects of simultaneous parameter variations can be understood using global sensitivity analysis, as was recently done for a model of acute inflammation [16]. Second, our model contained a simplified representation of the interaction between inhibitors and their targets. Specifically, we did not include the individual degradation/removal kinetics for the inhibitors, which reflects the assumption that the inhibitors are present at the inflammation site at nearly constant total concentrations. While this assumption may be only an approximation to the *in vivo* situation, it allowed us to focus our inhibition analysis specifically on the inhibitor-target interactions (rather than potentially complex inhibitor pharmacokinetics). Besides, an increase in the number of modeled components and their interactions would have caused an increase in the number of unknowns, thereby introducing additional uncertainty, which we wanted to avoid at this stage of the inhibitor modeling. Finally, our model did not explicitly represent the action of the recently emerged lipid mediators of inflammation resolution, such as lipoxins and resolvins [1, 17]. Yet, our modeling approach allowed us to implicitly represent their effects by modifying their suggested target mechanisms (see Figure 8 and Table II in [1]).

With the recognition of inflammation as the key contributor to several pathologies, new approaches are needed to identify mechanistic regulators of pathological inflammation. A promising methodology is based on the analysis of the inflammation indices that quantitatively characterize the shapes of inflammation trajectories. Our study shows the applicability of systems biology approaches to identify mechanistic regulators of the inflammation indices. Moreover, computational models can provide a non-invasive and cost-effective framework for testing the efficacy of potential therapeutic strategy aimed to improve inflammation resolution. Computational analyses employing robust control strategies can guide the development of focused, hypotheses-driven experimental and clinical studies by reducing the ambiguity in the timing and strength of therapeutic interventions.

## Materials and Methods

### Computational Model and Simulations

To simulate the inflammatory response, we used our recently developed quantitative model of acute and chronic local inflammation in a wound [11]. The model reflects initiation of inflammation by the platelets present at the site of injury; the platelets release TGF-β, whose gradient attracts inflammatory cells that interact and release soluble inflammatory mediators. The model’s variables describe the kinetics of 5 types of inflammatory cells (namely, active and apoptotic neutrophils, pro- and anti-inflammatory macrophages, and platelets) and 11 molecular mediators [namely, TNF-α, interleukin(IL)-1β, IL-6, IL-12, IL-10, CXCL8, TGF-β, platelet derived growth factor (PDGF), macrophage inflammatory protein-1α and 2 (MIP-1α, MIP-2), and interferon gamma-induced protein 10 (IP-10)]. We chose to model these components because they are widely regarded as essential cell types and molecular mediators involved in an innate immune response to injury [48, 75]. In our analysis, we did not include inflammatory molecular mediators produced by cells of adaptive immunity (e.g., IL-4 and IFN-γ) that are reported to affect innate immune components, such as macrophages [40]. For modeling tractability, we reduced the wide spectrum of possible wound macrophage phenotypes [34, 40] to just two: pro-inflammatory (similar to the “classically activated,” or M1, phenotype induced *in vitro* by IFN-γ and bacterial LPS) and anti-inflammatory (similar to the “alternatively activated,” or M2, phenotype induced *in vitro* by IL-4 and IL-13) phenotypes. The essential mechanisms (i.e., chemotaxis and phenotype conversion of inflammatory cells, cellular apoptosis, and molecular mediator production/degradation and positive/negative feedback effects) that govern the kinetics of the inflammatory response are represented via 69 model parameters (Table 1). The model describes extracellular signaling between different cell types and cytokines. Intracellular processes, such as transcription, translation, and export of proteins, are not represented mechanistically and are instead described implicitly via the model’s rate parameters.

Our model reflects experimentally observed acute and chronic inflammatory response kinetics initiated by injury or infection (see Figures 3 and 5 in [11]). While the kinetic trajectories of the model’s output variables in our simulations had qualitatively similar shapes during both acute and chronic inflammation, the chronic inflammatory scenarios were characterized by higher concentrations (Ψ_max_) and delayed resolution timing (R_i_) for neutrophils, macrophages, and pro-inflammatory mediators (e.g., TNF-α, IL-1β, and IL-6) in comparison with acute inflammatory scenarios, which is consistent with experimental reports of chronic inflammation [17, 34, 36, 45]. The model is a coupled system of 15 ordinary differential equations and one delay differential equation (DDE). The DDE in the model is used to describe the chemotaxis of pro-inflammatory macrophages. Indeed, there exists a ~12 h delay between the arrival of the macrophage precursors (i.e., monocytes) at the wound site and their differentiation into pro-inflammatory macrophages [12], which is accounted for in the model by using the DDE. Each of our simulations reflected a 20-day period after inflammation initiation. We performed all computations in the software suite MATLAB R2012a (MathWorks, Natick, MA) and solved the model equations using the MATLAB solver DDE23 with default tolerance levels. The MATLAB files used to simulate the results reported in this article and a document providing details on how run the code are provided as S1 Code and S1 Text, respectively.

### Computing Kinetic Trajectories and Inflammation Indices

For each model output variable representing a molecular species or cell type (with two exceptions), we calculated the four inflammation indices defined in the Introduction (Fig 1). We did not calculate the indices for the model variables representing the platelet and TGF-β concentrations, because their kinetic trajectories do not have the same characteristic single-peak shape as the trajectories for other model variables. We additionally considered, and computed the inflammation indices for, two variables representing the total concentrations of neutrophils (N_tot_) and macrophages (M_tot_). We computed these variables directly from the model output variables representing two distinct neutrophil and two distinct macrophage phenotypes. We thus analyzed 16 model variables in total. In the article text, we sometimes refer to the total neutrophil and total macrophage model variables simply as neutrophils and macrophages, respectively. First, we calculated temporal trajectories and the inflammation indices for the model’s default parameter set, which represents an acute inflammatory scenario [11]. Then, applying a previously described Latin hypercube sampling approach [71], we generated 10,000 random 69-parameter sets, in which each individual parameter was sampled independently from an interval permitting up to twofold deviations (up or down) from the parameter’s default value. We used the MATLAB function LHSDESIGN to perform this sampling. The parameter sets were intended to represent the natural variability in the inflammatory scenarios occurring under different circumstances or in different individuals. In the existing literature, there is no consensus regarding the criteria for selecting the parameter randomization sample size to ensure sufficient coverage of the possible kinetic scenarios. It is recommended that this sample size be increased until no significant further changes in the main analysis results are detected [76]. Following this strategy, we arrived at the parameter randomization sample size equal to 10,000 parameter sets. For the considered model variables, we calculated the inflammation indices from the temporal trajectories generated for each of these 10,000 parameter sets. We refer to these trajectories as 10,000 simulations.

### Local Sensitivity Analysis

We calculated logarithmic local sensitivities, *s*_*ij*_, for each inflammation index of every model output variable with respect to every model parameter according to the standard definition (see, e.g., [11, 73]):

$$s_{ij}=\partial logX_{i}/\partial logp_{j}=\left(dX_{i}/X_{i}\right)/\left(dp_{j}/p_{j}\right),$$ (1)

where *X*_*i*_ is a given inflammation index (T_act_, Ψ_max_, R_i_, or R_p_) for the model’s *i*th variable (of the 16 output variables) and *p*_*j*_ is the model’s *j*th parameter (of the model’s 69 parameters). To obtain numeric approximations of the derivatives in Eq 1, each parameter was individually perturbed by ±1% of its value, and the derivative was approximated using the second-order central finite difference formula. We restricted our attention to local sensitivities because local sensitivity analysis is a powerful tool that has been successfully used to identify critical mechanisms and intervention points in a variety of biological systems [11, 19, 72–74]. We performed a local sensitivity analysis for the default parameter set, as well as for each of the 10,000 simulations with random parameter sets. For each considered parameter set, each model variable, and each inflammation index, we sorted the absolute sensitivity values in descending order to determine the top three most influential parameters for that variable’s index.

### Correlation Analysis

Using the MATLAB function CORR, we calculated Spearman’s rank correlation coefficients (CCs) between each of the 69 model parameters and each of the four inflammation indices, for each of the 16 model variables. For these calculations, we used the model parameter values and the inflammation index values from the 10,000 simulations with randomized parameters described above. Moreover, based on the calculated R_i_ values for the neutrophil and macrophage variables, we introduced a criterion for dividing the 10,000 simulations into two subsets. One of the subsets contained simulations representing acute inflammation, and the other one contained simulations representing chronic inflammation. To create these subsets, we first calculated 20,000 ratios by dividing the R_i_ values for neutrophils and macrophages in each of the 10,000 simulations by the respective R_i_ values calculated using the default parameter set. Second, we used a cutoff value (equal to 2) to identify the simulations (out of 10,000) for which both the neutrophil and macrophage ratios were above the cutoff. The criterion for choosing the cutoff value was based on experimental studies, in which the R_i_ was increased by ~2-fold in abnormal inflammatory scenarios [12, 17]. The simulations separated based on this criterion were regarded as reflecting chronic inflammation. We then re-calculated the CCs between the model parameters and model output timing indices (i.e., T_act_ and R_i_) using only the chronic inflammation simulations. This was done to identify any significant correlations that may exist for a specific chronic inflammation condition and were not detected when all 10,000 inflammation scenarios (simulations) were considered. A description of the algorithm for the separation of the simulation subsets is provided in S1 Text.

### Inflammatory Mediator Inhibition Modeling

We modeled inflammatory mediator inhibition by adding two new differential equations to the original model for each modeled inhibitor. The equations represented the volumetric concentration of the inhibited mediator and its respective mediator-inhibitor complexes. We used mass action kinetics to model the individual reactions between the inhibitors and their target mediators according to the following reaction scheme (which reflects inflammatory mediator sequestration that prevents the mediator’s participation in normal signaling):

I + C  ←koff→  kon   IC (2)

where *C* denotes a mediator, *I* represents an inhibitor, and *IC* denotes the mediator–inhibitor complex. For any given inhibitor, *k*_*on*_ and *k*_*off*_ denote the values for the association and dissociation rate constants, respectively. Using our model extended in this way, we performed simulations to predict the effects of mediator inhibition at different inhibition concentrations and the addition of inhibitors at different time points after inflammation initiation. In these analyses, the inhibitors were added only in chronic inflammation simulations, and only one mediator was inhibited in each simulation, unless stated otherwise. A detailed description of the computational implementation of inflammatory mediator inhibition is provided in the Supplemental Material (S1 Text).

## Supporting Information

S1 Table Association (*k*_*on*_) and dissociation (*k*_*off*_) rate constants for the inhibitors of tumor necrosis factor α (TNF-α), transforming growth factor β (TGF-β), and the chemokine CXCL8. (PDF)

S1 Fig Neutrophil and macrophage concentrations in the case of TGF-β inhibition by different inhibitor concentrations added at different time points. Green and red lines represent computational predictions for acute and chronic inflammation, respectively. In all the subplots, the times of inhibitor addition (represented by the black lines) are as follows: dotted, 24h; dashed, 48h; and solid, 72h. Shown are normalized neutrophil (**a**-**c**) and normalized macrophage (**d**-**f**) kinetics for TGF-β inhibitor concentrations of 1 nM (**a** and **d**), 20 nM (**b** and **e**), and 200 nM (**c** and **f**). All the model-predicted values were normalized to the respective maximum values from the chronic inflammation simulations.
(TIF)

S2 Fig Sign of calculated correlation coefficients. Black and white colors represent positive and negative signs of the correlation coefficients (CCs), respectively. Subplots **a** and **c** show the CC sign for T_act_ and R_i_, respectively. Subplots **b** and **d** show the CC sign for Ψ_max_ and R_p_, respectively. The *x*-axis shows the model parameters designated by number (P#) (see Table 1 for a list of all model variables and parameters).
(TIF)

S3 Fig Correlation analysis for the model parameters and the inflammation indices of the model output variables. Black and white colors represent the index-parameter correlation coefficient (CC) values ≥0.5 and <0.5, respectively, for T_act_ (**a**), Ψ_max_ (**b**), and R_p_ (**c**). The CCs were calculated by performing the correlation analysis on 40,000 simulations, where the parameter values in each simulation were randomly selected from a 9-fold variation rage (i.e., 3-fold down, 3-fold up) around the default parameter values. The *x*-axis shows the model parameters designated by number (P#) (see Table 1 for a list of all model variables and parameters).
(TIF)

S1 Text Supplemental results and methods. (PDF)

S2 Text MATLAB code user manual. (PDF)

S1 Code MATLAB code. (ZIP)
