# Supplementary material for: Computational Identification of Mechanistic Factors That Determine the Timing and Intensity of the Inflammatory Response
Source: PLoS Comput Biol. 2015 Dec 3;11(12):e1004460. doi: 10.1371/journal.pcbi.1004460 (PMC4669096; doi:10.1371/journal.pcbi.1004460)
Supplement: S1 Table — (PDF) [file pcbi.1004460.s001.pdf]

**S1 Table.** Association ($k_{on}$) and dissociation ($k_{off}$) rate constants for the inhibitors of tumor necrosis factor α (TNF-α), transforming growth factor β (TGF-β), and the chemokine CXCL8.

| Inhibited cytokine | $k_{on}$ ($nM^{-1} \cdot h^{-1}$) | $k_{off}$ ($h^{-1}$) | Ref. |
|---|---|---|---|
| TNF-α | 0.93 | 4.69 | [20] |
| TGF-β | 13.35 | 0.00123 | [77] |
| CXCL8 | 3.25 | 0.830 | [78] |

## References


77. De Crescenzo G, Pham PL, Durocher Y, O'Connor-McCourt MD. Transforming growth factor-beta (TGF-beta) binding to the extracellular domain of the type II TGF-beta receptor: receptor capture on a biosensor surface using a new coiled-coil capture system demonstrates that avidity contributes significantly to high affinity binding. J Mol Biol. 2003;328: 1173-1183.
78. Yang XD, Corvalan JR, Wang P, Roy CM, Davis CG. Fully human anti-interleukin-8 monoclonal antibodies: potential therapeutics for the treatment of inflammatory disease states. J Leukoc Biol. 1999;66: 401-410.
