# Supplementary figures and images for: Computational Identification of Mechanistic Factors That Determine the Timing and Intensity of the Inflammatory Response
Source: PLoS Comput Biol. 2015 Dec 3;11(12):e1004460. doi: 10.1371/journal.pcbi.1004460 (PMC4669096; doi:10.1371/journal.pcbi.1004460)

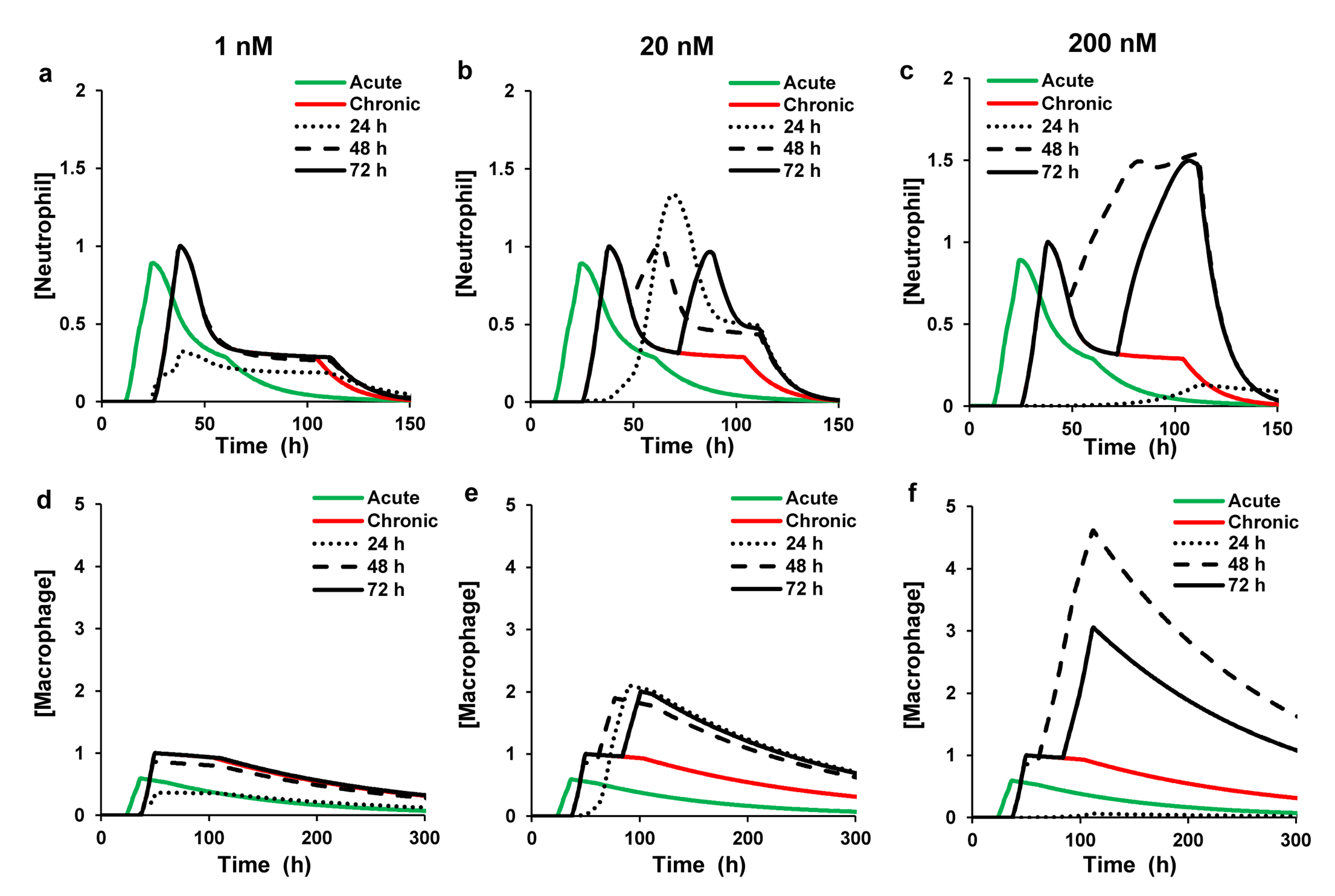

Supplement: S1 Fig — Green and red lines represent computational predictions for acute and chronic inflammation, respectively. In all the subplots, the times of inhibitor addition (represented by the black lines) are as follows: dotted, 24h; dashed, 48h; and solid, 72h. Shown are normalized neutrophil (a-c) and normalized macrophage (d-f) kinetics for TGF-β inhibitor concentrations of 1 nM (a and d), 20 nM (b and e), and 200 nM (c and f). All the model-predicted values were normalized to the respective maximum values from the chronic inflammation simulations. (TIF) [file pcbi.1004460.s002.tif]

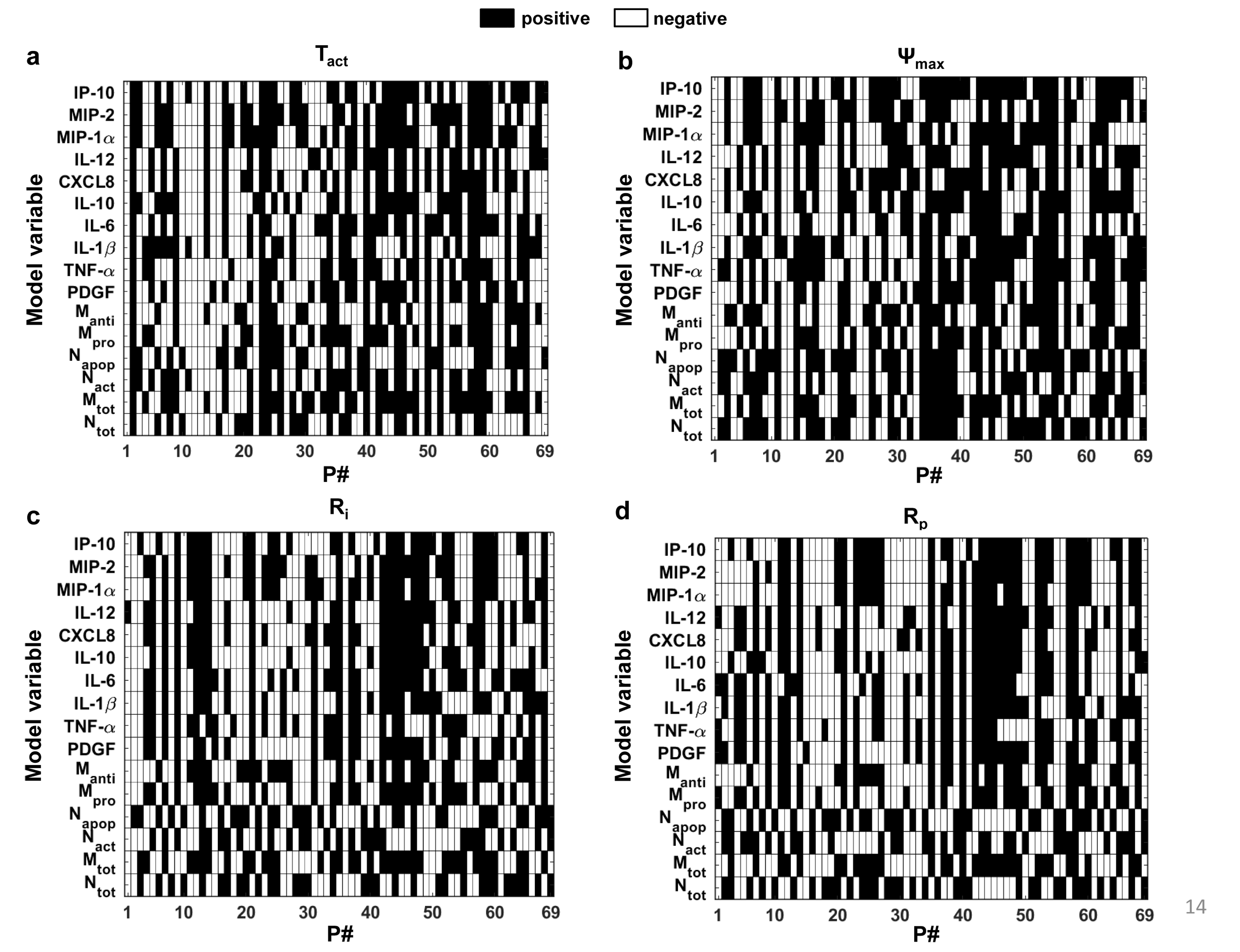

Supplement: S2 Fig — Black and white colors represent positive and negative signs of the correlation coefficients (CCs), respectively. Subplots a and c show the CC sign for Tact and Ri, respectively. Subplots b and d show the CC sign for Ψmax and Rp, respectively. The x-axis shows the model parameters designated by number (P#) (see Table 1 for a list of all model variables and parameters). (TIF) [file pcbi.1004460.s003.tif]

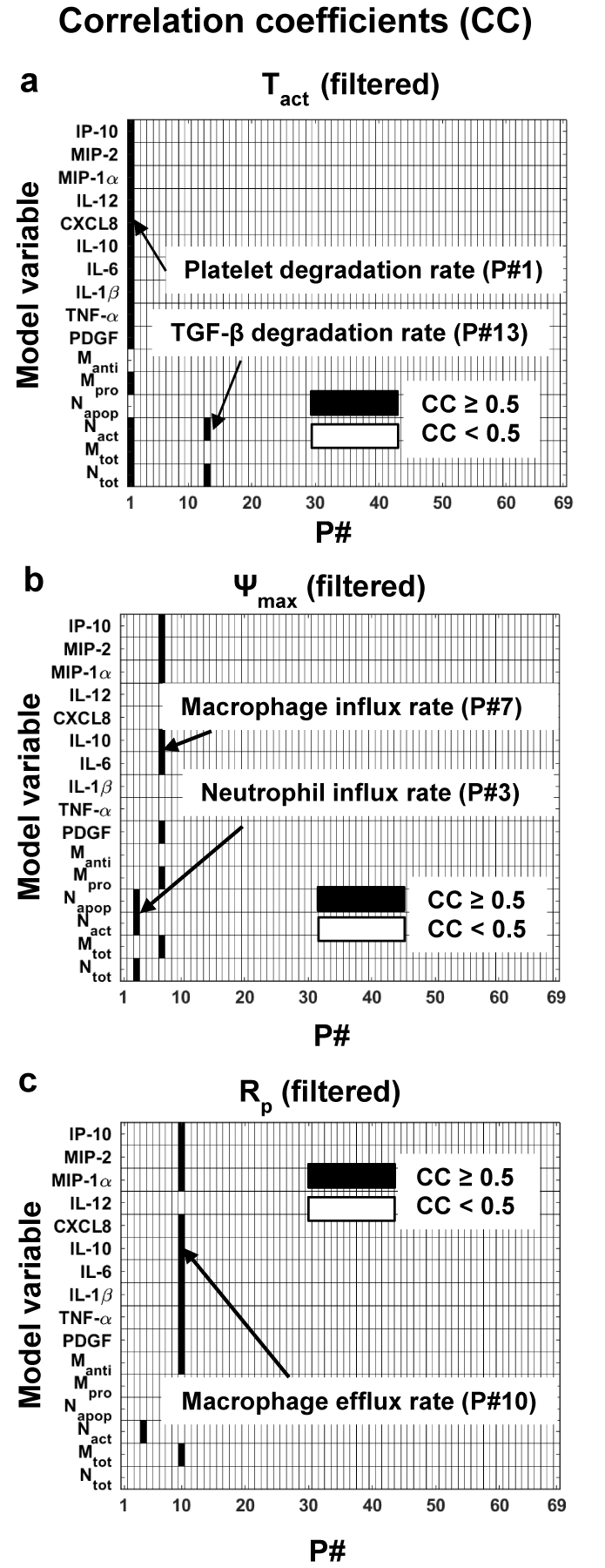

Supplement: S3 Fig — Black and white colors represent the index-parameter correlation coefficient (CC) values ≥0.5 and <0.5, respectively, for Tact (a), Ψmax (b), and Rp (c). The CCs were calculated by performing the correlation analysis on 40,000 simulations, where the parameter values in each simulation were randomly selected from a 9-fold variation rage (i.e., 3-fold down, 3-fold up) around the default parameter values. The x-axis shows the model parameters designated by number (P#) (see Table 1 for a list of all model variables and parameters). (TIF) [file pcbi.1004460.s004.tif]
