# Supplementary material for: Computational Identification of Mechanistic Factors That Determine the Timing and Intensity of the Inflammatory Response
Source: PLoS Comput Biol. 2015 Dec 3;11(12):e1004460. doi: 10.1371/journal.pcbi.1004460 (PMC4669096; doi:10.1371/journal.pcbi.1004460)
Supplement: S1 Text — (PDF) [file pcbi.1004460.s005.pdf]

S1 Text. Supplemental Results and Methods

Supplemental Results

In our simulations, TGF-β inhibition often restored total neutrophil and total macrophage Ψ_{max} (S1 Figure). Similarly to CXCL8 and TNF-α inhibition, when the added TGF-β inhibitor concentration was 1 nM, it was most effective in regulating the Ψ_{max} for both total neutrophils and total macrophages when added at 24 h (S1 Figure, a and d, dotted lines). However, other aspects of the kinetics of inflammation index regulation by TGF-β inhibition were different from those for TNF-α and CXCL8 inhibition. First, the concentration of the TGF-β inhibitor needed for restoration (at least, partial) of neutrophil or macrophage Ψ_{max} to their "normal" (i.e., acute-inflammation) values was in the range of ~1-10 nM (S1 Figure, a and d). These concentrations were at least an order of magnitude less than the effective inhibitor concentrations for TNF-α and CXCL8 (>100 nM, see Fig. 5). Second, when the TGF-β inhibitor was added at concentrations >10 nM, the T_{act}, R_i, and Ψ_{max} values for total neutrophils (S1 Figure, b-c) and the T_{act} and Ψ_{max} values for total macrophages (S1 Figure, e-f) often exceeded their respective values in the chronic inflammation trajectories in the absence of inhibitors. This unexpected and undesired effect resulting from a higher concentration of the TGF-β inhibitor may be explained as follows. TGF-β is the most powerful chemoattractant for both neutrophils and macrophages, with active concentrations in the picomolar range [79, 80], while TNF-α and CXCL8 display chemoattractant activity in nanomolar concentration ranges [81, 82] . Therefore, it is conceivable that, when higher concentrations of the TGF-β inhibitor are used, the free TGF-β levels can drop to the range of higher chemotactic activity (i.e., the picomolar range), thereby increasing the influx of neutrophils and macrophages into the inflamed site.

Supplemental Methods

Algorithm for the division of the 10,000 simulations into "acute" and "chronic" simulation subsets

The following sequence of two steps describes our algorithm for dividing the 10,000 simulations into the "acute" (i.e., representing acute inflammation) and "chronic" (i.e., representing chronic inflammation) subsets.

Step 1. For each simulation, we calculated the ratios of the R_i for the N_{tot} and M_{tot} model output variables to the corresponding R_i values for the default parameter set (representing acute inflammation). Thus, each simulation in the 10,000-simulation set was characterized by two ratios, one for N_{tot} and one for M_{tot}, which we denote Ratio(N_{tot}) and Ratio(M_{tot}), respectively.

Step 2. We defined a threshold Thr = 2. Then, for each simulation, we compared Ratio(M_{tot}) and Ratio(N_{tot}) with Thr. Those simulations for which Ratio(M_{tot}) ≥ Thr and Ratio(N_{tot}) ≥ Thr were designated as "chronic," whereas all other simulations were designated as "acute."

The threshold value Thr = 2 was chosen based on the experimentally observed differences between normal (i.e., acute) and abnormal inflammation [34, 45].

MATLAB implementation of the cytokine inhibition analysis at different time points

The simulations where the inflammatory mediator inhibitors were added at different time points were performed differently from the simulations where they were added at inflammation initiation (t = 0). In the latter type of simulations, the model equations were solved only once using the MATLAB solver DDE23 for the entire time span of our simulation, i.e., 20 days. In contrast, to implement the addition of cytokine inhibitors at time points other than t = 0, we solved the system of model equations twice (i.e., on two adjacent time intervals). The first run of the DDE23 solver, starting at t = 0, solved the system with the inhibitor concentration set to zero. The temporal end point for this first run was the selected time point at which the inhibitor was to be added to the system (i.e., 24 h, 48 h, or 72 h). Once the first run was complete, the values of all model variables at the final time point were used as initial values for the second run of the DDE23 solver. For this second run, the initial concentration of the TNF-α or CXCL8 inhibitor was set to 200 nM (Fig. 6). In the case of TGF-β, we used three distinct inhibitor concentrations, specifically, 1 nM, 20 nM, and 200 nM (S1 Figure). The temporal end point for the second run of the DDE23 solver was the final time point of our simulation, i.e., 20 days. Finally, the outputs from both runs were combined after removing the one duplicated time point, i.e., the time of the inflammatory mediator inhibitor addition.

References

79. Brandes ME, Mai UE, Ohura K, Wahl SM. Type I transforming growth factor-beta receptors on neutrophils mediate chemotaxis to transforming growth factor-beta. J Immunol. 1991;147: 1600-1606.
80. Wahl SM, Hunt DA, Wakefield LM, McCartney-Francis N, Wahl LM, Roberts AB, et al. Transforming growth factor type β induces monocyte chemotaxis and growth factor production. Proc Natl Acad Sci U S A. 1987;84: 5788-5792.
81. Pai R, Ha H, Kirschenbaum MA, Kamanna VS. Role of tumor necrosis factor-alpha on mesangial cell MCP-1 expression and monocyte migration: mechanisms mediated by signal transduction. J Am Soc Nephrol. 1996;7: 914-923.
82. Wang JM, Sherry B, Fivash MJ, Kelvin DJ, Oppenheim JJ. Human recombinant macrophage inflammatory protein-1 alpha and -beta and monocyte chemotactic and activating factor utilize common and unique receptors on human monocytes. J Immunol. 1993;150: 3022-3029.
