# Supplementary material for: Computational Identification of Mechanistic Factors That Determine the Timing and Intensity of the Inflammatory Response
Source: PLoS Comput Biol. 2015 Dec 3;11(12):e1004460. doi: 10.1371/journal.pcbi.1004460 (PMC4669096; doi:10.1371/journal.pcbi.1004460)
Supplement: S2 Text — (PDF) [file pcbi.1004460.s006.pdf]

MATLAB Code Description

5 October 2015

Computational identification of mechanistic factors that determine the timing and intensity of the inflammatory response

by Sridevi Nagaraja, Jaques Reifman, and Alexander Y. Mitrophanov

Correspondence about modeling- and software-related technical questions to Sridevi Nagaraja: snagaraja@bhsai.org

CONTENTS

Overview.........3

System Requirements.........3

MATLAB Code Description.........4

Inflammation index calculation.........5

Extended Sensitivity Analysis (randomized parameter sets).........6

Correlation Analysis (randomized parameter sets).........7

Molecular mediator inhibition model.........8

Overview

The MATLAB code for the inflammation model was developed at the Biotechnology High Performance Computing Software Applications Institute (BHSAI), Ft. Detrick, Maryland, to study the mechanistic determinants of the timing and amount properties of inflammatory cells and molecular mediators during normal and abnormal (delayed) inflammation. The model is further used to study the effect of molecular mediator inhibition on the kinetics of inflammatory cells and molecular mediators. The software currently implements a set of ordinary differential equations (ODEs) and a delay differential equation (DDE) to simulate acute and chronic inflammatory responses. Sensitivity and correlation analyses were implemented to identify the mechanistic regulators for the amount and timing indices of inflammatory cells and mediators. The inputs for the acute inflammation model as well as the sensitivity analyses are embedded in the code. The user can directly change parameter values inside some of the provided MATLAB files. The System Requirements Section contains the details about the computer system that we used to develop and run the code. The rest of this document provides information about using the code for the three different types of analysis described in the paper (inflammation index calculation for acute and chronic inflammatory scenarios, sensitivity and correlation analyses, and modeling of cytokine inhibition).

System Requirements

We used the following software and hardware components:

Software
• Operating System: Windows 7 Enterprise (64-bit operating system)
• MATLAB version 7.14.0.739 (R2012a) (64-bit operating system)
• MATLAB Statistics Toolbox, Version 8.0 (R2012a)
• Microsoft Excel 2010 for plotting the figures in the paper

Hardware
•Intel® Core(TM) 2Duo CPU E8400 @ 3.00 GHz and 4.00 GB RAM
• Disk space: 3–4 GB is recommended for a typical installation
For extended sensitivity analysis, we used two server computers with the following specifications:
• CPU: 2x Intel Xeon X5650 (6 cores @ 2.66 GHz)
• RAM: 24 GB @ 1333 MHz
• Disk space: 4x 300 GB @ 10,000 RPM

MATLAB Code Description

The code was developed in MATLAB 2012a. It includes the following files:

**Main.m**	–	simulation routine that runs the acute inflammation model and calculates the inflammatory indices for all model output variables
**Main_inhibition.m**	–	simulation routine that runs the molecular mediator inhibition model
**Main_timeinhibition.m**	–	simulation routine that runs the molecular mediator inhibition model for inhibitor introduction at different user specified time points
**inflammation_delay.m**	–	function comprising model equations, as well as chemotaxis and cytokine feedback functions
**inflammation_delay_inhibition.m**	–	function comprising the additional equations to model molecular mediator inhibitors
**Parameters.m**	–	script containing parameter values and initial conditions (also contains the program's main INPUT)
**Parameters_inhibition.m**	–	script containing parameter values and initial conditions including activation and deactivation rate constants for inhibitors of the molecular mediators TNF-α, TGF-β, and CXCL8
**Graphs_main.m**	–	script that performs basic plotting of all model variables
**Curvecharacteristic.m**	–	function for calculating the logarithmic local sensitivity values for each inflammatory index for each model output variable for a given parameter set
**Global_local.m**	–	simulation routine for extended sensitivity analysis (in the vicinities of 10,000 random parameter sets)
**timescalculation.m**	–	function for calculating the four inflammatory indices for all model variables
**Correlation_Spearman.m**	–	function for calculating the spearman's correlation coefficient and their respective p-values between the 10,000 parameter values and 10,000 index values for each index for each model output variable
**CC_figure.m**	–	function for plotting the results of the correlation analysis

Latinhypercube.m – function that generates a user-defined number of random parameter sets

Instructions for downloading and saving the MATLAB files

The files are currently available in a zip folder that can be downloaded from the POLS computational Biology website. In order to run them in MATLAB, please follow the instructions given below:
1. Download the zipped folder S1 Code onto your system.
2. Unzip the folder and save all the files into a new folder.
3. Open folder created in step 2 in MATLAB as your current working folder.

Inflammation index calculation

1. The **INPUT** to the model is the **initial concentration of platelets** which reflects the severity of an injury. The default value of this parameter is **2×10^8 platelets/mL**. This value is defined in the **"Parameters.m"** file under **"Initial conditions"**. To increase or decrease the severity of an injury, increase or decrease the value of the parameter **"P_init"** in this file.

Note: Any additional simulation of interest, e.g., chronic inflammation induced by a 5-fold higher macrophage influx rate or by an alteration in the platelet degradation rate, will need to be initiated by changing the respective parameter in the **"Parameters.m"** file.

2. To run the model, open "**Main.m"** and click the **"Run"** icon 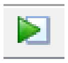 or type **"Main"** in the MATLAB command window.

3. After the routine is executed, to display all model variables run **"Graphs_main.m"**.

4. These output figures show the raw output values calculated by the model. The raw values of all output variables, as well as simulation time points, are stored in the output variable **"g"** in the MATLAB workspace.

5. The values of the four inflammatory indices for all model output variables are stored in the output variable "**Q1**" in the MATLAB workspace as well as individually in the output variables **"AT"**, **"DT"**, **"PH"**, and **"RP"**.

Note: The raw values of all output variables of the model were imported into Microsoft (MS) EXCEL, normalized, before being used in the figures in the manuscript.

Note: Chronic inflammation simulations shown in Fig. 4 of the paper were performed by executing the file **"Main.m"** after increasing the macrophage influx rate parameter **"kM_in"** by 5-fold of its default value in the **"Parameters.m"** file.

Note: Chronic inflammation simulations shown in Figs. 5 and 6 of the paper were performed by executing the file **"Main.m"** after increasing the macrophage influx rate parameter **"kM_in"** by 2-fold of its default value, reducing the platelet degradation rate parameter **"kdP"** by 2-fold of its default value, and increasing the initial platelet concentration **"P_init"** by 100-fold of it default value in the **"Parameters.m"** file.

Extended Sensitivity Analysis (randomized parameter sets)

Simulation: To calculate sensitivity values for a number of random parameter sets, follow the instructions given below:

1. Open **"Global_local.m"**.

2. Change the value of the parameter **"iter"** to choose the number of randomly generated parameter sets (default value used in the paper: 10,000).

3. Change the value of the parameter **"rangefactor"** to increase or decrease the uniform distribution sampling range for the extended sensitivity analysis [default value used in the paper: 2, i.e., the parameter values in the generated sets are chosen randomly from a 4-fold range (2-fold higher and 2-fold lower than default value)].

Note: To perform the sensitivity analysis on one parameter set using one node takes approximately 5 minutes. Therefore, to compute the sensitivity values for 10,000 parameter sets, we used 12 parallel nodes, and the simulation took approximately 16 hours to complete. The user is recommended to start with smaller values of **"iter"** (e.g., 50 or 100). If using parallel processing, replace the **"for"** command in **"Global_local.m"**, line 30, by **"parfor"** and specify the number of nodes that will be used in **"matlabpool"** command right before using **"parfor"**.

4. Click on the **"Run"** icon or type **"Global_local"** in the MATLAB command window.

Output: The raw sensitivities for the four inflammatory indices (T_{act}, Ψ_{max}, R_i, and R_p) for all the model outputs with respect to all the 69 parameters for the 10,000 random parameter sets are stored in the output variables **"G_ATf"**, **"G_PHf"**, **"G_DTf"**, and **"G_RPf"** in the MATLAB workspace. The actual values of the inflammatory indices calculated for all the model output variables for the 10,000 parameter sets are stored in the output variables **"ATf"**, **"PHf"**, **"DTf"**,

and **“RPf”**. The size of each of these matrices is 18x10,000. The values of the model parameters in the 10,000 sets are stored in the output variables **“Param1”**. These output arrays are saved in one output variable called **“outputvars.mat”** and are further used for the calculation of the correlation coefficient between the model parameters and inflammation indices of model output variables. The user may individually save the output variables once the run is complete.

Note: For a complete list of the parameter identifying numbers, check the file **“Parameters.m”**. Each parameter is assigned an identifying number shown in the comment next to its initialization and in Table 1 of the paper.

5. Table 2 show the identifying number of parameter whose sensitivity had a given rank (highest, second highest …) for the largest fraction of the 10,000 randomly generated parameter sets for each of the selected output variables (in our case: total neutrophils, total macrophages, TNF-α, IL-1β, IL-6, and IL-10). The actual value of this largest fraction is provided in the braces in Table 2.

Correlation Analysis (randomized parameter sets)

Simulation: To calculate the Spearman’s rank correlation coefficient values between each model parameter and each model output variable, follow the instructions given below:

1. Load the saved output variable **“outputvars.mat”**, or individually load the saved inflammatory index values calculated during the extended sensitivity analysis, i.e., **“ATf.mat”**, **“DTf.mat”**, **“PHf.mat”**, **“RPf.mat”**, and **“Param1.mat”** output variables into the MATLAB workspace.

2. Open **“Correlation_Spearman.m”**.

3. Click on the **“Run”** icon or type **“Correlation_Spearman”** in the MATLAB command window.

4. For plotting the correlation coefficients, download the file **“format_ticks.m”** from MATLAB central file exchange (http://in.mathworks.com/matlabcentral/fileexchange/15986-format-tick-labels).

5. Open **“CC_figure.m”**.

6. Click on the **“Run”** icon or type **“CC_figure”** in the MATLAB command window.

Output: The correlation coefficient values for the 4 inflammatory indices are stored in 4 separate output variables **“RHO_AT”**, **RHO_RI”**, **“RHO_PH”**, and **RHO_RP”**. The figure generated using **“CC_figure.m”** only generates a color map of the correlation values. The x-axis and y-axis labels were entered manually.

6. To identify the model parameter subsets representing chronic inflammation, uncomment and run the last section of the MATLAB file **"Correlation_Spearman.m"**. For completing this part of the simulation, download the file "istersectm.m" and " ismember.m" from MATLAB central file exchange (http://www.mathworks.com/matlabcentral/fileexchange/28341-set-functions-with-multiple-inputs/content/SetMI/intersectm.m and http://www.mathworks.com/matlabcentral/fileexchange/28341-set-functions-with-multiple-inputs/content/SetMI/ismemberm.m)

Molecular mediator inhibition model

Simulation: Two types of simulations were performed with respect to molecular mediator inhibition.

a) Varying the concentration of the molecular mediator inhibitor introduced in the system at time = 0 (Refer to Fig. 5 of the paper).

b) Varying the time points (24 h, 48 h, and 72 h) at which molecular mediator inhibitors were introduced in the system at a concentration of 200nM for TNF-α and CXCL8 inhibitors (Refer to Fig. 6 of the paper)and at a concentration of 1nM, 20 nM, and 200 nM for TGF-β inhibitor (Refer to S1 Figure of the paper).

In the paper, all the simulations with molecular mediator inhibitor action are simulated along with the parameter changes that simulate chronic inflammation in the model.

<u>a) Instructions for running the code for varying the molecular mediator inhibitor concentration</u>

1. Open the **"Parameters_inhibition.m"** file and introduce the following changes.
To induce chronic inflammation, increase the macrophage influx rate parameter **"kM_in"** by 2-fold of its default value, reduce the platelet degradation rate parameter **"kdP"** in by 2-fold of its default value, and increase the initial platelet concentration **"P_init"** by 100-fold of it default value.

2. To introduce the individual/combined inhibition of a particular molecular mediator in the model, change its/their initial value(s) in the **"Parameters_inhibition.m"** (Itgf_init, Itnf_init, or IIL8_init)

3. Save the file with the changed model parameter values in steps 1 and 2.

4. Open the file **"Main_inhibition.m"**.

5. Click on the **“Run”** icon or type **“Main_inhibition”** in the MATLAB command window.

Output: The inflammatory time course of all model variables are stored in the output variable **“Y1”**, and their respective inflammatory index values are stored in the output variable **“Q1”** in the MATLAB workspace.

Note: The raw values of all output variables of the model were imported into Microsoft (MS) EXCEL, normalized, before being used in the figures and tables in the manuscript.

b) Instructions for running the code for introduction of the molecular mediator inhibitors at different time points

1. Open the **“Parameters_inhibition.m”** file and introduce the following changes.
To induce chronic inflammation, increase the macrophage influx rate parameter **“kM_in”** by 2-fold of its default value, reduce the platelet degradation rate parameter “**kdP**” in by 2-fold of its default value, and increase the initial platelet concentration “**P_init**” by 100-fold of it default value. Make sure that the initial values for the three molecular mediator inhibitors are set to zero. (Itgf_init = 0, Itnf_init = 0, IIL8_init = 0). Save the file with the changed model parameter values.

2. Open the file **“Main_timeinhibition.m”**.

3. Select the time points at which a particular molecular mediator inhibitor is to be introduced by changing the values in the model variable **“dosetime”** (Line 19 in the MATLAB file). For the simulations in the paper, we have used the time points of 24 h, 48, h and 72 h.

4. Depending on the individual/combination of the molecular mediator inhibitor(s) that need to be introduced, uncomment the corresponding lines (lines 45-47) and (lines 58-60) in the file **“Main_time inhibiton.m”** as described below:

For TGF-β (line 45 and line 58)
yinit2(17) = 5 (use a value between 1nM -10 nM)
and
F_TGF{k} = [g1(1:dosetime(k),:);g2];

For TNF-α (line 46 and line 59)
yinit2(19) = 200 (use a value between 100 nM -500 nM)
and
F_TNF{k} = [g1(1:dosetime(k),:);g2];

For CXCL8 (line 44 and line 60)
yinit2(21) = 200 (use a value between 100 nM -500 nM)
and
F_CXCL8{k} = [g1(1:dosetime(k),:);g2];

5. Click on the **"Run"** icon or type **"Main_timeinhibition"** in the MATLAB command window.

Output: The inflammatory time course of all model variables are stored in the output variables **"F_TGF"**, **"F_TNF"**, or **"F_CXCL8"** in the MATLAB workspace, depending on which of the three molecular mediators is being inhibited. These output variables are of type structure of size 1x3 where each element of the structure contains the inflammatory kinetics for all model variables for a given time point when the molecular mediator was introduced in the system.

Note: The raw values of all output variables of the model were imported into Microsoft (MS) EXCEL, normalized, before being used in the figures and tables in the manuscript.
